# Macromolecular networks and intelligence in microorganisms

**DOI:** 10.3389/fmicb.2014.00379

**Published:** 2014-07-22

**Authors:** Hans V. Westerhoff, Aaron N. Brooks, Evangelos Simeonidis, Rodolfo García-Contreras, Fei He, Fred C. Boogerd, Victoria J. Jackson, Valeri Goncharuk, Alexey Kolodkin

**Affiliations:** ^1^Department of Molecular Cell Physiology, Vrije Universiteit AmsterdamAmsterdam, Netherlands; ^2^Manchester Centre for Integrative Systems Biology, The University of ManchesterManchester, UK; ^3^Synthetic Systems Biology, University of AmsterdamAmsterdam, Netherlands; ^4^Institute for Systems BiologySeattle, WA, USA; ^5^Molecular and Cellular Biology Program, University of WashingtonSeattle, WA, USA; ^6^Luxembourg Centre for Systems Biomedicine, University of LuxembourgEsch-sur-Alzette, Luxembourg; ^7^Departamento de Bioquímica, Instituto Nacional de CardiologíaMexico City, Mexico; ^8^Department of Automatic Control and Systems Engineering, The University of SheffieldSheffield, UK; ^9^School of Computer Science, The University of ManchesterManchester, UK; ^10^Netherlands Institute for NeuroscienceAmsterdam, Netherlands; ^11^Russian Cardiology Research CenterMoscow, Russia; ^12^Department of Medicine, Center for Alzheimer and Neurodegenerative Research, University of AlbertaEdmonton, AB, Canada

**Keywords:** microbial intelligence, emergence, decision-making, robust adaptation, association, anticipation, self-awareness, problem solving

## Abstract

Living organisms persist by virtue of complex interactions among many components organized into dynamic, environment-responsive networks that span multiple scales and dimensions. Biological networks constitute a type of information and communication technology (ICT): they receive information from the outside and inside of cells, integrate and interpret this information, and then activate a response. Biological networks enable molecules within cells, and even cells themselves, to communicate with each other and their environment. We have become accustomed to associating brain activity – particularly activity of the human brain – with a phenomenon we call “intelligence.” Yet, four billion years of evolution could have selected networks with topologies and dynamics that confer traits analogous to this intelligence, even though they were outside the intercellular networks of the brain. Here, we explore how macromolecular networks in microbes confer intelligent characteristics, such as memory, anticipation, adaptation and reflection and we review current understanding of how network organization reflects the type of intelligence required for the environments in which they were selected. We propose that, if we were to leave terms such as “human” and “brain” out of the defining features of “intelligence,” all forms of life – from microbes to humans – exhibit some or all characteristics consistent with “intelligence.” We then review advances in genome-wide data production and analysis, especially in microbes, that provide a lens into microbial intelligence and propose how the insights derived from quantitatively characterizing biomolecular networks may enable synthetic biologists to create intelligent molecular networks for biotechnology, possibly generating new forms of intelligence, first *in silico* and then *in vivo*.

## INTRODUCTION

For centuries, mankind has grappled with the precise nature and defining features of intelligence. Debates have erupted over how to define and measure the extent of intelligence in parts of the biological (and non-biological) world. Alan Turing, for example, famously proposed a test for evaluating the performance of “artificial intelligence”: namely, can it be distinguished from the performance of human beings by another human (Turing, 1950)? There have also long been philosophical discussions on what can be considered “intelligent.” A number of studies have explored whether there are differences in intelligence between human populations (Neisser et al., 1996), whether animals (Thorndike, 1998), and even plants (Trewavas, 2002) exhibit intelligent behaviors, whether non-human artificial systems are capable of intelligence (Brooks, 1991) and, more recently, whether intelligence spans biological domains including even the simplest of microbes (Hellingwerf et al., 1995; Bruggeman et al., 2000; Hoffer et al., 2001; Ben Jacob et al., 2004). For the purposes of this discussion, however, and in the interest of brevity, we limit ourselves to systems of biological nature.

As an abstract concept, “intelligence” escapes easy definition. As a linguistic construct, its characteristics have varied substantially across philosophical and cultural contexts. Here, we do not attempt a definition of intelligence; rather, we discuss how some features (like decision-making) commonly associated with a brain can also be found in the microbial world. Rather than launch an ontological, epistemological, or semantic inquiry, we instead focus on the scientific utility of assigning intelligence to microbes. We review how the mathematical perspectives of complex adaptive systems and recent data-intensive developments in systems biology offer insight and help structure this problem. Finally, we consider whether viewing microbes through the lens of “intelligence” can help us better describe their behavior, harness their intelligence to perform valuable actions and, in the end, possibly extend our understanding of the systems biology underlying the functions of the human brain.

### WHAT IS “INTELLIGENCE”?

The modern biological perspective on “intelligence,” even at its most fundamental level, tends to associate it with the human brain. In this context, “intelligence” is a property of the human brain, or a feature that somehow emerges from its activity. Accepting that intelligence may not be exclusively a feature of the human brain, but rather it may be present – at least to a degree – in all creatures possessing brains or nervous systems, already helps refine the general features of intelligence. However, intelligence may not have to be associated solely with a certain biological organ, such as a brain or a nervous system. Brains and nervous systems may be highly adapted conduits for expressing and integrating multiple intelligent behaviors. Some of these behaviors may be exhibited by other complex adaptive systems present in living organisms that do not have a brain or nervous system. As early as 1995, Hellingwerf et al. (1995) suggested that some two-component systems in bacteria comply with the requirements for elements of a neural network. More recently, the so-called biogenic approach of cognition has gained momentum by focusing on the biological origin of cognition and intelligence, abandoning a strict anthropocentric perspective (Lengeler, 2000; Lyon, 2006; van Duijn, 2012). This is the central paradigm around which we base our analysis.

### HOW DOES INTELLIGENCE EMERGE?

A small molecule at room temperature cannot be intelligent; it cannot store information about its past with implications for its behavior in some future. Large macromolecules, such as proteins and polynucleotides, may store information as, for example, Gibbs free energy in metastable states, where interactions between their structural components can differ depending on the way they were folded some time ago. The primary difference between small and large molecules with respect to information storage is that small molecules have a sufficiently small number of structural microstates (i.e., conformations) such that all of these states are visited by the molecule on time scales relevant for biochemistry (∼10 ms), i.e., they are “ergodic” (Westerhoff and Van Dam, 1987). However, large molecules may not visit all of their microstates, even on equivalent or greater time scales. In principle, phosphorylation, dephosphorylation and other chemical modifications may increase the possible number of microstates (Kamp and Westerhoff, 1986). High energy nucleic acid and protein complex states called chromatin, for example, may take hours, if not days, to relax after refolding.

Information storage within an object requires that the object be away from its equilibrium state for a sufficient period of time. This can be achieved transiently by bringing the object into a high free energy state, with the relaxation back to the equilibrium state being slow. Or, it may be achieved permanently by making this process permanent (at the cost of Gibbs free energy), such as in the terminal phosphoryl bond in ATP. More generally, in open systems, Gibbs free energy harvested from the environment can be used to maintain the non-equilibrium state. Such free-energy transductions require non-linear interactions of multiple components: they require complexity (Westerhoff and Van Dam, 1987) – and so does intelligence.

*Vis-à-vis* memory, intelligence is an emergent property of a complex system; a feature that is not reducible to the parts of the system in isolation. Intelligence emerges when system components interact. For example, the intelligence (or intelligent-like behavior) we observe inside a single cell emerges from interactions among thousands of non-intelligent macromolecules. Similarly, the intelligent behavior of a microbial society is not simply the sum of the behavior of intelligent cells; rather, it is a property that emerges from the interactions amongst many of them. In the human brain, intelligence emerges from interactions of nearly 90 billion neurons.

While, in practice, it is not trivial (or yet possible) to specify the interactions leading to intelligence, a promising start would be to catalog all of the interacting components (molecules, microorganisms, neurons), thereby defining the topology of the interactions as a network. Experimentally, this would correspond to performing Chip-on-chip, yeast two-hybrid experiments or antibody pull-down experiments. However, as we will show, this does not suffice to establish a basis for intelligence. It is not the mere existence of a network that begets intelligent behavior – a rock can be full of networked structures in the form of bonds among its component molecules and ions, yet it is not intelligent. Rather, it is the dynamics of the interactions in a system that generate the system-level property we call intelligence. Somehow, non-linearities in the interactions and their indirect and incomplete, yet non-zero, reciprocities are important.

Although we have discovered many of the components of living systems, e.g., neurons and their connectivity in the brain (Alivisatos et al., 2012; Ahrens et al., 2013) and macromolecules and their interactions in the cell, we still have no clear view on how they collectively contribute to intelligence. One reason for this failure is that the complete picture may be too complex to be perceived fully by our human brains. With computer simulation, however, it should be possible to reconstruct the emergence of these properties. Even then, it is debatable whether our brain, biased by its very human nature, will be able to identify and appreciate all forms of intelligence, especially those that are dissimilar to our own. Identifying unfamiliar forms of intelligence is the transcendental challenge of this paper – one that would have enormous implications for synthetic biology and engineering. We start by describing features of microbial systems that are analogous to familiar forms of human intelligence.

## SYSTEMS BIOLOGY OF INTELLIGENCE: RECONSTRUCTING THE EMERGENCE OF INTELLIGENCE FROM COMPONENT PROPERTIES OF THE SYSTEM

Systems biology can be defined as a science that aims to understand how biological *function* that is absent from macromolecules in isolation *emerges* when these macromolecules exist as components of a *system* (Alberghina and Westerhoff, 2005; Westerhoff et al., 2009). The concepts of System, Function and Emergence are central in this context.

The notion of function plays an important role in (systems) biology. Yet, often this concept is ill-defined. Because the word “*function*” has strong teleological connotations, many biologists hasten to clarify that they invoke neither purpose nor intention when they use the notion of function. The subtle reasoning that accompanies these notions, however, is often overlooked (Wouters, 1999; Looijen, 2000), not in the least because the term “*function*” is used in various ways. Here, we adopt the perspective of Wouters (1999), who distinguished four principal kinds of biological function. In short, he argues that the term “*function*” is used to refer to: (i) function as activity; (ii) function as role; (iii) function as advantage; and (iv) function as selected effect (Wouters, 2003, 2013). Mahner and Bunge (2001) arrived independently to a similar set of functions. Considering the “cognitive” functions that are discussed in this study (decision-making, robust adaptation, association, anticipation, self-awareness and problem solving), the first three definitions are the most useful. The fourth definition is used in evolutionary biology and it features in historical evolutionary explanations.

Defining “*function*” is important to understand the explanations of biological systems we craft. We need, for instance, to distinguish mechanistic explanations and design explanations. Mechanistic explanations categorize a system into a number of functional components; they describe how these components are arranged, how their activities are organized in time, and relate these features to some phenotype (Boogerd et al., 2013). Mechanistic models are mathematical models related to the activities of cellular reaction networks involving transport, metabolism, signal transduction, or gene expression. However, mechanisms only suffice to explain how the features are brought about (how they work). Understanding *why* certain mechanisms exist (rather than other, alternative organizations) requires design explanations (Wouters, 1995, 2007). These explanations typically contrast observed organizations with conceivable alternatives in an attempt to identify invariances (or “laws”) that can account for our observations. Delineating the difference between these two types of explanations relates to how we attribute function to systems (e.g., “function as an activity” versus “function as an advantage”).

A human brain comprised by neurons, a microbial community comprised by different species and individual organisms or an individual cell comprised by molecules are all semi-open systems. They all selectively interact with their environments by way of mass and energy exchange, where the decrease of free energy in the environment is coupled to the increase of the order of the biosystem itself (decreasing its own entropy), or with the maintenance of the biosystem against the activity of the many processes that tend to dissipate it (Westerhoff and Van Dam, 1987). Systems of artificial intelligence are semi-open as well. They all need an external energy source to maintain their existence. In other words, there is always a flow of mass and energy through the system, and then a certain function emerges.

The function in which we are interested here is “*intelligence*.” Intelligence consists of many features that allow a system to adapt to its environment. Together with other functions of the system, intelligence emerges from interactions among system components. As an emergent property, it satisfies three theses, as expounded by Stephan: (i) physical monism; (ii) synchronic determinism; and (iii) systemic (organizational) property (Stephan, 1999). The thesis of physical monism restricts the nature of the system’s elements and states, so that the system consists of only physical entities and interactions, denying any supernatural influences – this is how we describe our system *ab initio*: we neglect all supernatural influences *de juro*. The thesis of synchronic determinism restricts the way systemic properties and the system’s microstructure are related to each other and states that there can be no difference in systemic properties without changes in the structure of the system or in the properties of the components: features of intelligence are underlined exactly by the changes in the system (firing between neurons, chemical reactions between molecules, electrical current between components of a computer); in other words, differences in systemic properties should be measurable at least in principle and, with the advent of genomics and the other -omics, also in practice. It is noteworthy that this thesis also implies that the inverse statement is invalid: a change in a system’s microstructure or properties does not necessarily yield a change in its behavior or properties. The thesis of being a systemic property means that a property is not exhibited by elements in isolation; interactions must keep the elements out of their non-informative equilibrium state.

If emergence is weak, it simply satisfies just the three theses stated above. According to Stephan (Stephan, 1999, 2006), strong emergence would satisfy one additional criterion – *irreducibility*. In general, there are three conditions for irreducibility, but it has been argued that for biochemical networks only one condition is relevant (Boogerd et al., 2005): if the properties of parts (say A, B, and C) in their relationship (R_ABC_) *within* the system as a whole (together constituting an explanation of the systemic property at hand) do not follow from the properties of parts (A, B, C) or simpler subsystems (AB, BC, AC) in isolation, it is a strongly emergent property. It should be noted that in this definition of strong emergence, the deduction base does not include systemic knowledge, such as the state of the system. Cognitive-like capabilities of a single microbial cell might then be irreducible in the sense that these properties cannot be deduced from the full knowledge of the behavior of the parts of the system in isolation or in configurations simpler than the one prevailing within the whole system. In fact, all features of microbial intelligence described in this study are expected to be irreducible in this sense, and therefore strongly emergent.

It is worthwhile to compare our notion of strong emergence with that from philosophy of mind. In philosophy of mind, mental properties like human intelligence are considered strongly emergent; contrary to our contention here, however, the underlying reason for this limitation is that the property does not follow from the behavior of the parts and their interactions within the system. By contrast, we assert that microbial intelligence, or in principle any systemic property, can be mechanistically explained if the properties and behaviors of the parts and their relationship within the system are fully known, i.e., when full knowledge of the state of the system is available. For this reason, any microbial property can, in principle, be mechanistically explained and, thus, can also be reconstructed in mathematical models of the underlying mechanism provided that knowledge of the system is fully available. Properties that are declared strongly emergent – because of a limited deduction base – are still calculable if the behavior of all relevant components and their mutual interactions within the system are available (Boogerd et al., 2005).

The limited deduction base of strong emergence provides the opportunity to rank emergent systemic properties according to the strength of emergence, which can be clarified as follows: in principle, every single component of the system, albeit indirectly, interacts with all other components. Let us consider an example of two abstract proteins A and B binding to each other inside the cell. The binding reaction between proteins A and B might depend on the presence of other proteins. For example, transporters and structural proteins forming intracellular compartments keep proteins A and B together or separate. Other proteins (e.g., chaperones) might modulate the interaction directly by chemical modification of the interacting proteins. Binding between proteins A and B can also depend on environmental parameters, like intracellular pH. However, the pH is the result of proteins that regulate the uptake and pumping out of ions and different buffering molecules. In turn, ion transport processes are coupled to ATP hydrolysis and thus are dependent on the Gibbs free energy flux through the cell. Thus, the interaction between two components in the cell depends to a variable extent on the state of the whole system. In other words, system component properties are state dependent. The greater their state dependency is, the greater the degree of irreducibility of the system (non-deducibility), implying stronger emergence (Kolodkin et al., 2012a,b).

The ability of a system to “choose the best option to solve a question and to anticipate the future” and, thus, to be intelligent might be state-dependent to a very high extent. Nevertheless, the intelligent response can be reconstructed in a computer model if we have complete knowledge of the properties of and the interactions between all components in the system. Similarly to other forms of emergence, intelligent behavior is somehow predetermined by the system itself and by applied stimuli. Theoretically, with precise mathematical description of all system components, all interactions among the components and with appropriate boundary conditions, the emergent intelligent behavior reconstructed with a model should become an accurate description of the manifestation of intelligence of a real system. But in practice, we do not possess the extremely accurate information necessary to model a real system precisely, because there is a large degree of uncertainty involved in measuring or even acquiring all system parameters, or the extreme complexity of the system makes it difficult to understand or even know the mechanisms of all system processes. So, intelligent response may not be 100% reproducible in a simulation, not because of the “free will” of the system, but rather because of the limitations of our current knowledge and abilities.

A description of how components interact with and affect each other can be represented as a network: metabolic networks, signal transduction networks, gene expression networks, anatomical networks, microbial ecological networks, etc. One can generate and model these networks using various approaches. For example, one can determine the kinetic rules of how network components interact and express the rates of these interactions in terms of mathematical relationships, e.g., differential equations. Then, one can integrate all equations and solve them for the whole system. As a result, one may be able to simulate the dynamic behavior of the network and, thus, reconstruct its emergent properties *in silico*. For example, the response of the nuclear receptor network to the cortisol signal has been modeled in a kinetic ODE-based model (Kolodkin et al., 2013a). The intelligent properties of the physiological network were reconstructed in the computer model; for instance, the modeled system was able to learn from previous stress and anticipate the next cortisol pulse.

The example above shows how intelligent behavior can emerge from just one feedback and one feedforward loop. In reality, the network can be much more complicated and contain many such loops. Biologically inspired “intelligence” models and algorithms have been extensively developed in the fields of artificial intelligence and optimization with many real-world applications, such as artificial immune systems (Smith and Timmis, 2008), evolutionary algorithms, artificial neural networks (Rolls and Treves, 1998) and the Kirdin kinetic machine (Gorbunova, 1999). For instance, feedback and feedforward loops, based on the architecture of neurons (including synapses and dendrites), are crucial for understanding the functional connectivity in the brain that is usually modeled by the artificial neural networks (Rolls and Treves, 1998). Neural networks are mainly classified into two groups, i.e., (i) the feedforward neural networks (FFNNs) where data is propagated from input to output using “combinatorial machines,” e.g., radial basis function (RBF), multilayer perceptron (MLP), self-organizing map (SOM); and (ii) the recurrent neural networks (RNNs). Several important feedforward loop motifs have been identified in both neuronal connectivity networks and transcriptional gene regulation networks (Milo et al., 2002), despite these networks operating on different spatial and temporal scales. This similarity in motifs may reflect a fundamental similarity in the evolved designs of both types of networks: to reject transient input fluctuations/noises and activate output only if the input is persistent, a so-called persistence detector (Alon, 2007). In addition, a multi-input feedforward structure is identified in the neuronal network of the nematode *Caenorhabditis elegans*, which serves as a so-called coincidence detector: the output is activated only if stimuli from two or more different inputs occur within a certain period of time (Kashtan et al., 2004; Alon, 2007). Another biological example appears in the retina, where a hierarchical feedforward cortical architecture is used for the pre-processing of visual information (Sanger, 1989). Although successful in practical applications, pure FFNNs are expected to be rare in the human neural system. On the other hand, RNNs have immediate biological application (i.e., self-organizing dynamic systems) and can describe complex non-linear dynamics, including both feedforward and feedback structures. Nevertheless, very few real applications have been studied based on RNNs. Until recently, RNNs have been employed to study short-term memory and brain-like memory (Salihoglu, 2009). This is because RNNs allow the output of a neuron to influence its input, either directly or indirectly, via its effect on other neurons. This allows the network to reflect the input presented to it, but also its own internal activity at any given time. In intracellular macromolecular network organization, a distinction has been made between dictatorial and democratic hierarchies, where only in the latter case the metabolite concentrations close to the systems output are able to influence gene expression (Westerhoff et al., 1990; Snoep et al., 2002). The two types of hierarchy may affect FFNNs and RNNs, respectively.

Learning and memory are two important, counterposed features of “intelligence.” The former assimilates new information, requiring flexibility in the network to produce complex dynamics; the latter retains old information, requiring stability in the network with sufficient storing capacity. Tradeoffs between the two can be modeled and observed using neural networks. A recent study (Hermundstad et al., 2011), for example, investigated the relationship between the neural network architecture (e.g., parallel and layered networks) and performance mediated through FFNNs. Another study (Salihoglu, 2009) indicated that classical feedforward networks with gradient descent learning algorithms are not sufficient to describe complex memory and learning dynamics, because real brain dynamics (e.g., memory) are more complex than fixed point attractors, i.e., characterized by cyclic and chaotic regimes. Hence, classical feedforward networks with gradient descent learning algorithms may not converge when complex non-linear dynamics (e.g., bifurcation) exist. In this case, RNNs may be a more appropriate choice for describing memory-like structures. In addition, feedback structures can increase network stability and exhibit the paradoxical property of near-perfect adaptation, where many properties of the system remain constant even when the system is subject to an environmental challenge or strong change in other network properties (He et al., 2013).

**FIGURE 1:**
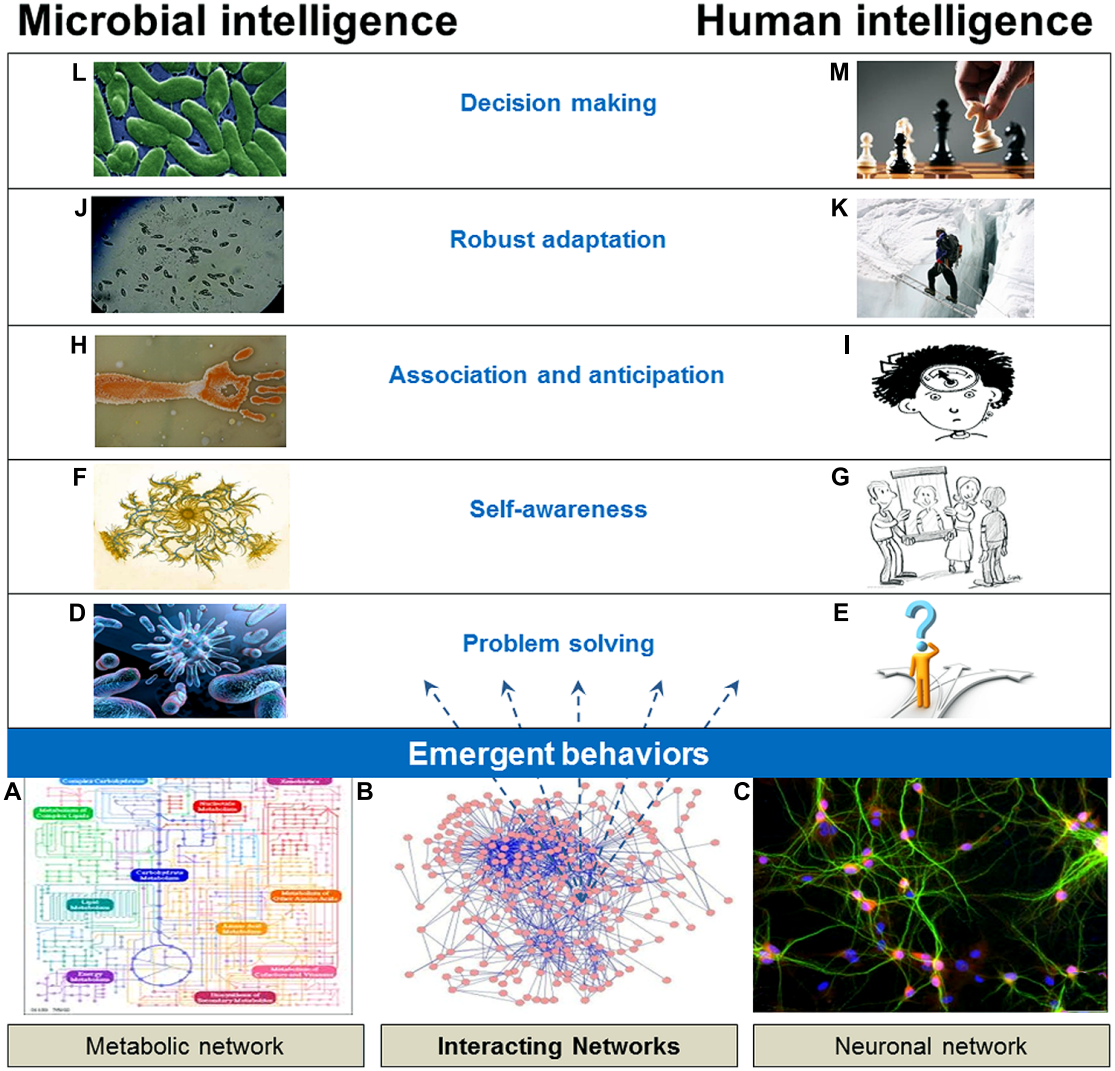
**Microbial intelligence vs. human intelligence.** Microbes exhibit similar characteristics of intelligence as higher organisms and humans, such as decision making, robust adaptation, association and anticipation, self-awareness and problem solving capabilities. **(A)** Overview of metabolism as a molecular circuit; Taken from www.genome.ad.jp/kegg/kegg.html on September 23, 2002; **(B)** Supplementary Information for: “Topological network alignment uncovers biological function and phylogeny” from bio-nets.doc.ic.ac.uk/home/software/graal/; **(C)** Hippocampal Neurons, from learn.fi.edu/learn/brain/proteins.html; **(D)** www.humanillnesses.com; **(E)** Problem solving, from www.kaizen-factory.com; **(F)** tamar.tau.ac.il/~eshel/html/Bacteria_art_gallery.html; **(G)** www.getupanddosomething.org; **(H)** Baumel bacterial cartography, from www.nextnature.net; **(I)** shperspectives.wordpress.com; **(J)** Infusoria, from ikanrainbowfish.blogspot.com/2013/07/kultur-infusoria.html; **(K)** Sherpa mountaineer crossing the Khumbu icefall – Wikimedia Commons en.wikipedia.org/wiki/File:Pem_dorjee_sherpa_(2).JPG; **(L)** www.nyas.org/image.axd?id=a0d6067c-80c6-4a30-9a8d-3c319b199796&t=633845693265270000; **(M)** Chess, from corporate-games.ro.

These examples provide a high-level overview of how to reconstruct and understand the emergence of intelligence using information about component relationships, even when intelligence is strongly emergent. In the next section, we refine our understanding of intelligence in microbes by detailing examples of microbes exhibiting specific characteristics of intelligence (**Figure 1**).

## MANIFESTATIONS OF INTELLIGENCE IN THE MICROBIAL WORLD

### DECISION-MAKING

Decision-making in humans is a vital process undertaken on a daily basis. It is a complex process that involves the coordinated activity of an extended neural network, including several different areas of the brain. Making a decision requires the execution of several subtasks, such as outcome appraisal, cost–benefit analysis, and error perception, before finally selecting and implementing the optimal action. These processes can also be influenced by several factors such as personal preference, reward evaluation, reinforcement learning and social cooperation (Assadi et al., 2009; Gleichgerrcht et al., 2010). In the microbial world, decisions are made by monitoring the current state of the system, by processing this information and by taking action with the ability to take into account several factors such as recent history, the likely future conditions and the cost and benefit of making a particular decision. At the population level, microbes are also capable of hedging their bets, by having individuals of an isogenic population in different states even when experiencing the same environmental conditions, and they are also able to make collective decisions that cause the entire population to respond in a particular way. Microbes are able to make decisions based on different criteria of information and also to perform the decision-making using different mechanisms, utilizing different types of molecular networks.

It can be argued that even simple biological systems like viruses are capable of decision-making when interacting with their host under certain conditions. A well-studied example is the bacteriophage lambda lysis/lysogeny decision upon infection of *Escherichia coli*. The decision is regulated at the genetic level by a bistable switch, formed by mutual repression (Wegrzyn and Wegrzyn, 2005). The decision is made based on the conditions of the host cell and the number of phages present. However, stochastic effects are also thought to play a role, either through stochasticity in the expression and regulation of the lambda switch system (Arkin et al., 1998) or through differences between host cell environments prior to infection (St-Pierre and Endy, 2008). The fact that microbes experience stochasticity, due in part to low molecule numbers and the probabilistic nature of molecular interactions, adds layers of complexity to the decision-making process, for example the need to discriminate between signal and noise. With relatively recent technological advances, experimental measurements of stochasticity are more readily obtained and it has been found to affect some decision-making systems. This should be of no surprise, as stochasticity is at the basis of all time dependent processes – high molecule numbers and linearity being the forces that remove stochasticity from observation (Westerhoff and Van Dam, 1987).

One of the earliest known systems where a microbe makes decisions is that of ammonia transport and assimilation in *E. coli* (van Heeswijk et al., 2013). The ammonium transporter (AmtB), the ammonium assimilating enzymes glutamate dehydrogenase (GDH) and glutamine synthetase (GS), and the helper enzyme glutamate synthase (GOGAT) are the main players in ammonium transport and assimilation at low environmental ammonium availability. A decision needs to be made between high-cost, high-accumulation transport by AmtB, low-cost, low-affinity assimilation by GDH, and high-cost, high-affinity assimilation by GS/GOGAT. In making this decision, *E. coli* balances several tradeoffs: (i) maintaining intracellular ammonium at levels sufficient for growth; keeping in check energy costs (ii) of transport and (iii) of assimilation; (iv) minimizing a futile cycle generated by ammonium-ammonia movement across the membrane; and (v) preventing or minimizing the wastage of ATP by the simultaneous action of biosynthetic GS and degradative GDH. This delicate decision is made in *E. coli* through the action of a complex hierarchical regulatory network, simultaneously involving gene expression, signal transduction, metabolic regulation and transport (Kahn and Westerhoff, 1991; Bruggeman et al., 2005; Boogerd et al., 2011; van Heeswijk et al., 2013).

Many prokaryotic cells are able to move through liquids or over moist surfaces by using a variety of motility mechanisms (swimming, swarming, gliding, twitching, floating) and mostly use complex sensory devices to control their movements (Jarrell and McBride, 2008). The decision of microbes to move toward nutrient sources or away from toxic compounds is another observation that appears “intelligent.” The most studied system is that of chemotaxis in *E. coli*, with common features in other prokaryotes and eukaryotes. In order to make this decision, the cell monitors the environment by means of multiple receptors in the cell membrane. The information of the ligand binding to the receptor, and the processing of this information inside the cell, is achieved by means of a signaling pathway involving methylation and phosphorylation, as opposed to the genetic switch seen in the lysis/lysogeny decision (Bourret and Stock, 2002). The level of phosphorylated CheY, the downstream protein of the signaling pathway, determines which movements the cell undertakes: when phosphorylated CheY is bound to the flagellar motor (i.e., when an attractor ligand is present) it rotates counter-clockwise, resulting in a straight swimming movement; in the absence of phosphorylated CheY the unbound flagellar motor rotates clockwise, resulting in a tumbling motion. Using this mechanism, organisms make a biased-random walk, with the length of the periods of straight swimming dependent on the signal, resulting in movement toward or away from different stimuli.

*Pseudomonas aeruginosa* has been shown to make its decisions about which of its two siderophore-dependent iron acquisition systems to use when faced with iron limitation based on the cost-to-benefit ratios of the two options (Dumas et al., 2013). The two mechanisms have different costs and benefits to the cell: one mechanism, using the pyoverdine siderophore, has a high iron scavenging efficiency (since pyoverdine has a high iron affinity, *K_a_* = 10^24^ - 10^32^ M^-1^), but comes at a high cost, requiring the expression of at least 14 genes, hence utilizing high amounts of nucleotides, amino acids, ATP, and other cellular resources. The other mechanism, using the siderophore pyochelin, has a lower cost to the cell because of a reduced biosynthetic pathway consisting only of seven genes, hence requiring the utilization of few cellular resources, but has a much reduced efficiency of iron-acquisition (since its affinity to iron is relatively low, *K_a_* = 10^5^ - 10^6^ M^-1^). Here, information processing and decision making is achieved by the finely tuned parameters of the two systems’ feedback loops that enable them to exhibit different sensitivities. The parameters of the feedback loop for the high-cost, high-efficiency system limit its use to extreme iron limitation conditions and the parameters of the feedback loop for the low-cost, low-efficiency system enable it to be utilized in more moderate iron limitation, thereby optimizing the cost–benefit ratio.

The decision of *Bacillus subtilis* to become transformation-competent (i.e., able to take up DNA) is made at an individual level; yet, the mechanism by which it occurs results in a reproducible portion of the population making the decision to become competent. The decision making regulatory system is a bistable switch that operates near a critical threshold that, once passed, leads to a committed decision to become competent (Maamar et al., 2007; Leisner et al., 2008). Due to this system operating close to the threshold, stochastic fluctuations in the levels of one protein, ComK, are able to push the cell over the threshold to begin the transition to competence (Maamar et al., 2007). As this is based on stochasticity, it will only occur in a portion of the cells in a population. Since this results in different phenotypes from an isogenic population of cells in the same environment, it is considered to be a bet-hedging strategy (Veening et al., 2008). Although each individual may not be in the optimal state for the given conditions, the population as a whole gains an advantage by becoming more adaptable.

Through the above examples of decision-making in microbes, it can be seen that there are several common features that are analogous to processes involved in human decision-making. Although the network components may vary (gene-expression regulation, signaling pathways, metabolism, transport), the networks involved and the parameters controlling their interactions allow the microbes to monitor their environment, process the information and react, effectively making a decision in an “intelligent” manner by taking into account such factors as the cost–benefit ratio and population survival strategies. We note, however, that decision-making in microbes is not limited to the examples contained here. More importantly, the mechanisms for generating decision-making behaviors are not confined to the particular mechanisms described. Recent work aimed at constructing genome-wide protein interaction networks, for example, has revealed many additional molecules and interconnections that play important roles in these processes (Noirot and Noirot-Gros, 2004).

### ROBUST ADAPTATION

An important feature of “intelligence” in microbes is the robust adaptation to changes in environments. Such robust adaptions include homeostasis, as well as adaptive tracking of nutrient sources (Patnaik, 2000) and evasion of harmful compounds (e.g., bacterial chemotaxis, mentioned previously). Almost all adaptation mechanisms involve feedback or feedforward regulation structures (or motifs). These can be relevant for signaling, gene regulatory and metabolic networks, where homeostasis can be introduced via fine-tuning of rate constants in feedback and feedforward motifs. Relatively long-term adaptations often involve changes in genetic expression, such as gene mutations, transcription/translation activities or rewiring of gene regulatory networks – for a review see (Brooks et al., 2011). Examples include adaptation to salt conditions, temperature or asymmetric cell division. Short-term adaptation, on the other hand, typically involves regulation mediated by (i) protein–protein interactions and covalent modifications (e.g., phosphorylations) in signal transduction pathways; or (ii) allosteric or more direct substrate–product effects in metabolic networks. Of all the adaptive regulations, robust perfect adaptation is of particular interest. It describes an organism’s response to an external perturbation by returning state variables to their original values before perturbation. For example, perfect adaptation has been reported in bacterial (e.g., *E. coli*) chemotaxis (Berg and Tedesco, 1975; Alon et al., 1999; Yi et al., 2000; Hansen et al., 2008), osmotic-stress adaptations (Muzzey et al., 2009), and MAP-kinase regulation (Hao et al., 2007; Mettetal et al., 2008). Such perfect adaption behaviors are thought to be introduced through a time integral on the “controlled variable” in the network, which corresponds to a specific control system structure, i.e., an integral feedback control (Csete and Doyle, 2002). A recent *in silico* study (Ma et al., 2009) identified an alternative topology that can also ensure perfect adaptation through an incoherent feedforward structure, where a positive regulation cancels out the effect of a simultaneous negative regulation, hence the overall output is insensitive to the input signal. Because it has been difficult to experimentally discriminate between perfect and strong adaptation and because at least some of the proposed mechanisms for perfect adaptation require biochemically unrealistic features [including zero order degradation of proteins (He et al., 2013)], the evidence for truly perfect adaptation needs to be revisited. In many cases, adaptation may be less perfect, with robustness being strong, but limited. Here, it would help if robustness were quantified (Quinton-Tulloch et al., 2013). In non-robust “proportional” (He et al., 2013) regulations, the appearance of a specific signal or environmental condition can be a direct indicator/predictor of a particular response. The feedforward regulatory mechanism, then, is introduced to respond directly to the signal rather than to the disturbance. Feedforward regulatory structures were observed in gene regulatory networks in the regulation of membrane lipid homeostasis (Mangan and Alon, 2003; Albanesi et al., 2013), in bacterial photosynthesis genes for optimal free-energy supply (Mank et al., 2013), and in the heat shock response in *E. coli* (Shudo et al., 2003).

Different regulation mechanisms in living cells often occur at multiple levels simultaneously with a hierarchical structure (Westerhoff, 2008). For example, in a microbial metabolic network, the regulation of a reaction rate can be achieved by the modulation of (i) enzyme activity through a substrate or product effect, or through an allosteric effect, i.e., metabolic regulation; (ii) enzyme covalent modification via signal transduction pathway; or (iii) enzyme concentration via gene expression, gene-expression regulation. Such multi-level regulation corresponds to different control loops in a control system, which may ensure the robustness versus perturbations at various frequencies, as employed in engineering system design. Let us consider an unbranched metabolic pathway, with the first enzyme inhibited by the end-product via both allosteric/metabolic and gene-expression regulation. If the flux demand on the end-product module increases rapidly, the concentration of the end-product decreases rapidly. Often, as a result of the allosteric effect of the end-product directly on the first enzyme, the activity of that first enzyme increases quickly too. This metabolic control of enzyme activity is a fast “actuator” of the system. However, if there is a further increase in the flux demand, the first enzyme may lose its regulatory capacity since its activity may be approaching its maximum capacity (*k*_cat_). At this stage, the system has a second “adaptation” through gene expression that is slow but leads to an increase in the concentration of the first enzyme, which then decreases the direct stimulation of the catalytic activity of the first enzyme. The regulation of the first enzyme is then bi-functional in dynamic terms (Csete and Doyle, 2002): the metabolic regulation rapidly buffers against high frequency perturbations, but possibly with small amplitude or capability, while the gene-expression regulation is slow to adapt, but may be able to accommodate very large constant perturbations (Ter Kuile and Westerhoff, 2001).

When interpreting metabolic and gene-expression regulation separately as specific “control system structures,” the former was recently identified as more of a “proportional control” action (Yi et al., 2000; El-Samad et al., 2002) with limited range and the latter as more of an “integral control” action with potentially a wider range, but acting more slowly (He et al., 2013). Such control engineering interpretations can also be linked with classical Metabolic Control Analysis (MCA; Fell, 1997) and Hierarchical Control Analysis (HCA; Kahn and Westerhoff, 1991). The relatively fast metabolic regulation is related to the direct “elasticities” of MCA, while the slow gene-expression regulation corresponds to the indirect “elasticities” of HCA (He et al., 2013).

### ASSOCIATION AND ANTICIPATION

Associative learning allows one to model how two or more features in the world co-vary and respond accordingly. This type of learning provides context, in the sense that it specifies how several features in the environment, or within cells, change together. It implies that the learner has a mechanism to encode mutual information. In humans and animals, this type of learning has been associated with experimental settings where, for example, a subject is conditioned (often through an auditory or visual cue) to activate unconditioned responses (like salivation) after presenting the subject with a conditioned stimulus (e.g., a bell) simultaneous to the unconditioned stimulus (e.g., dinner) that usually elicits the unconditioned response. After a period of learning the association, the unconditioned response (salivation) can be achieved in the absence of the unconditioned stimulus (simply ringing the bell). Conditioned behaviors like this have been well studied in humans and other animals since the pioneering work of Ivan Pavlov (Pavlov and Anrep, 1927). Recently, the molecular mechanisms responsible for encoding these behaviors in neurons have been defined (Maren et al., 2013). In general, these mechanisms rely on the plasticity of neurons to reinforce electrochemical couplings, such as changing the localization and abundance of glutamate and NMDA receptors at synapses (Nakazawa et al., 2002; Rumpel et al., 2005). The development of recurrent artificial neural networks, for example Hopfield networks (Hopfield, 1982), has provided a computational model for studying the processes of associative memory.

Associative learning allows learners to structure dependencies that exist in the world. Pavlov’s dog, for example, salivates because of the linkage the dog has learned between bell and dinner; even though the association is entirely manufactured in this case. Outside of contrived laboratory conditioning, associative learning occurs when environmental variables are physically coupled, or somehow co-vary non-randomly. For example, the increase in the level of light (photons) at sunrise, signals associated changes in the environment, such as increase in temperature, change in O_2_ availability, etc. Organisms leverage these physical associations to better adjust their physiology in specific environments (Bonneau et al., 2007), to employ easily measured proxies as indications for other phenomena (like the bell for Pavlov’s dog) and, in some cases, even use the cues themselves to prepare or “anticipate” subsequent alterations to the environment. Investigators have asked recently whether organisms like microbes, which do not have nervous systems, can also exhibit associative learning and anticipation.

Several experimental studies and modeling efforts have suggested that, indeed, microbes can learn associations, both as communities and individually. Studies, furthermore, suggest that gene regulatory networks can encode associative learning. One of the most comprehensive examples of this phenomenon comes from a study of the bacterium *E. coli* (Tagkopoulos et al., 2008). As a microbe that lives both in the soil and the guts of mammals, *E. coli* has to adjust its physiology to environments that vary with respect to important biological parameters, such as temperature and oxygen availability. Since many of these environmental parameters do not change randomly, but rather in coupled ways (e.g., increase of temperature in the oral cavity and corresponding decrease in oxygen availability in the gut), *E. coli* is able to take advantage of this predictable physical association to direct its physiology accordingly. In this study, the authors demonstrated that transcriptional responses in elevated temperatures are highly similar to those observed in oxygen perturbation experiments, even though the second stimulus is absent (much in the same way that Pavlov’s dog can be stimulated to salivate simply by ringing a bell). More impressively, they showed that *E. coli* can “re-learn” these associations. Relative to ancestral *E. coli*, evolved strains grow better in environments where temperature and oxygen are decoupled (in this case inverted). This study demonstrates (1) that microbes have both the capacity for associative learning, and (2) that the learned associations are plastic. A similar study in yeast suggested that previous lifestyle plays an important role in adaptation to severe stress, re-emphasizing the existence of associative learning in microbes (Berry et al., 2011).

It is important to note, however, that time-scale for this “learning” is on the order of evolutionary processes and most likely involves genetic changes. This has an analogy in the development of “fixed” hard-wired neuronal connections in a brain or cultural learning in human society. In the example, it took many generations for *E. coli* to learn about the altered association between oxygen and temperature and, presumably, much longer for the natural situation to be canalized. Critical questions for future studies will include whether gene regulatory networks encode associations that are capable of being learned within the lifetime of an individual bacterium; a case in point was made for ammonia assimilation in *E. coli* (Hellingwerf et al., 1995; Bruggeman et al., 2000). A recent modeling study suggested that gene regulatory networks composed of bistable elements with stochastic dynamics can exhibit associative learning, although the number of learnable associations may scale as the square root of the number of bistable elements (Sorek et al., 2013). Similar results have been obtained in the context of chemical networks (McGregor et al., 2012) and other transcriptional networks (Carrera et al., 2012). Additional experiments, however, are required to evaluate whether the dynamics of cellular networks with multiple stable states are sufficient to encode and retrieve contextual associations. Hellingwerf et al. (1995) showed learning behavior should be possible in realistic mono-stable *E. coli* networks.

Among microbial populations, associative learning seems to be commonplace. Mechanisms and examples of associative learning in microbial communities have been discussed extensively elsewhere (Ben Jacob et al., 2004; Xavier, 2011). Typically, associative learning in microbial populations involves some sort of social communication (such as quorum sensing, discussed in see Quorum Sensing and Self-awareness in Microbial Populations and Communities). This type of networked communication is highly plastic and eminently reminiscent of neuronal activities. Other examples of association and anticipation in the microbial world are exhibited by pathogenic bacteria such as *P. aeruginosa*, which is an important human, animal, and plant opportunistic pathogen and, perhaps, the bacterial species that has most genes devoted to regulatory purposes (Stover et al., 2000). In the context of human digestive tract infections, this bacterium senses several compounds released by the host tissues, such as interferon, opioids, and metabolites like adenosine, which are all released into the intestinal tissues and lumen during surgical injury, ischemia and inflammation. In addition, it senses the extracellular levels of phosphorus, which decrease severely when the patient’s condition deteriorates. Hence, when the bacterium senses high concentrations of host-released compounds together with a decrease in phosphate levels, it anticipates the vulnerability of the patient and turns on several virulence determinants that frequently lead to lethal sepsis (Zaborin et al., 2009). Recently, it was demonstrated that in the genus *Burkholderia*, quorum sensing allows the activation of cellular enzymes required for production and secretion of oxalic acid, which serves to counteract ammonia-mediated alkaline toxicity during the stationary phase, hence anticipating a stress situation and triggering a preventive strategy that helps cells better adapt to the oncoming harsh environmental conditions (Goo et al., 2012).

The capacity for associative learning among microbes may be one of the reasons why we are able to reverse engineer them. Since microbes do not respond to stimuli independently, but rather their internal networks direct common responses to diverse but related environmental signals, regulatory networks in microbes can be reconstructed by simply measuring their response across a broad range of conditions. Gene regulatory networks, for example, can be inferred in three simple steps: (i) perturb cells across a broad range of relevant conditions; (ii) measure their transcriptional response in each environment; and (iii) cluster similar gene expression patterns observed reproducibly across environments. Mining for genetic similarities among genes sharing a particular expression pattern, such as common *cis-*regulatory elements in their promoter regions, in turn helps link these transcriptional modules to some of the molecular mechanisms responsible for regulating them. In practice, such approaches allow the construction of gene regulatory networks directly from transcriptome measurements (Reiss et al., 2006). It should be recognized, however, that the networks thus reconstructed are incomplete, as they forego the signal transduction and metabolic networks that are part of the actual regulation (Ter Kuile and Westerhoff, 2001).

### ASSOCIATIVE LEARNING IN PROTOZOA

Early investigation of intelligent traits in microbes, such as associative learning and memory, occurred in ciliated protozoa. While early studies concluded that ciliates are capable of associative learning, several experimental design flaws have led to skepticism about these conclusions. For example, Soest (1937) reported that the ciliate *Stentor* contracts if exposed to light after conditioning with simultaneous luminous stimuli and electrical shock. The author concluded that *Stentor* exhibited classical condition response; the study, however, lacked important controls, such as training *Stentor* with the administration of shocks alone (Corning and Von Burg, 1973). A similar study suggested that paramecia perform instrumental conditioning (Gelber, 1952). The author observed that paramecia attached preferentially to a bare wire that had been baited previously with bacteria compared to a wire that had not been baited. It was demonstrated later, however, that the behavior likely resulted from increased bacterial concentration near the wire rather than as a consequence of associative learning (Jensen, 1957). Even the paradigmatic example of learned escape from the bottom end of narrow capillary vertically positioned tubes into a larger volume of media by *Stentor* and *Paramecium* has been refuted. Subsequent to the initial report of this behavior, it was noticed that the strategy simply entailed decreased upward swimming. In fact, the same behavior was observed when the task was reversed, demonstrating that this behavior is unlikely to be the result of associative learning (Hinkle and Wood, 1994). Furthermore, fixing the capillary in a horizontal position could be a better experimental set-up for examining associative learning (Kunita et al., 2014). It should be noted, however, that these examples may have been insufficient to meaningfully test associative learning, since they did not reflect abilities required by protozoa in their natural environments.

Contemporary research has focused instead on ecologically salient intelligent behaviors, such as mate selection, foraging and hunting (Clark, 2010a,b, 2012, 2013). This new wave of research has renewed interest in ciliate intelligence. More significantly, it has reinforced the claim that ciliate protozoa indeed have remarkable learning abilities, including complex cooperation and competition behaviors usually attributed to higher organisms. The observations imply an ability to learn and adjust mating strategies using Hebbian-like associative learning behavioral heuristics.

The ciliate *Spirostomum ambiguum*, for example, learns to advertise mating fitness to suitors and rivals during the preconjugal courtship. Fitter suitors – “conspicuous consumers” – advertise their status by avoiding exchange of preconjugal touches, despite the metabolic cost of swimming away. Less fit individuals – “prudent savers” – on the other hand, wait for favorable opportunities for partner conjugation, conserving energy and exhibiting lower avoidance frequencies. Interestingly, both “conspicuous consumers” and “prudent savers” learn to switch between the two strategies, apparently tuning their behavioral heuristics and switching frequencies to optimize mate selection (Clark, 2010c).

Less fit individuals are even capable of “cheating” in this system. These individuals take advantage of a fit suitor’s “conspicuous consumer” behavior. A less fit individual positioned between a fit suitor and potential mate may, for example, corrupt the “conspicuous consumer’s” contraction-reversal movements (e.g., flip the signal from avoidance to conjugation). The “cheater” can physically interact with these signals, since they are spread as vibrations through viscous media. As a result, the “cheater” can conjugate with a mating partner that has been “aroused” by the fit suitor’s actions. The signal would be easy to take advantage of if it were scripted in a binary encoding (e.g., 0 – no contraction, 1 – contraction); however, suitors appear to encode a low probability of contracting and reversing simultaneously, in addition to simple contraction and reversal behaviors. This would make ciliate mating signals resemble a quantum bit flip channel used in quantum computing (Clark, 2010b). Encoding mating communication with a contraction-reversal qubit would make it far more robust to “cheating” behaviors of competitors.

Evolution of error-correction systems that counteract degradation of mating signals is quite remarkable. These mechanisms must account for non-random color noise created by mixing of vibrations emitted by mating rivals and suitors, as well as random ecological white noise (Clark, 2010b). It would seem that ciliates have developed coding schemes to diagnose, decrease, and counteract mating-signal errors due to noisy information processing (Clark, 2013).

These findings suggest that quantum computing concepts may be required to understand emergence of intelligent communication in microbes. Without the concept of qubits, for example, we would have been unable to describe the complex encoding of ciliate mate selection behaviors. Quantum computing was first proposed in the 1980s (Manin, 1980; Feynman, 1982), so one has to wonder how the expansion of our knowledge horizons may influence our understanding of intelligence in all forms of life in the future.

### QUORUM SENSING AND SELF-AWARENESS IN MICROBIAL POPULATIONS AND COMMUNITIES

Quorum sensing is a widespread type of bacterial cell–cell communication between individuals of the same or different species (Waters and Bassler, 2005; Lee et al., 2007; Hosni et al., 2011). The accepted paradigm for this kind of communication is that individual cells steadily produce and release several kinds of small diffusible molecules (signals), called auto-inducers. In parallel, each cell has the ability to sense the presence of those molecules, by means of receptors/transcriptional modulator proteins that bind the auto-inducers and, once complexed with them, trigger a global transcriptional response that leads to crucial changes in the expression of several phenotypes and behaviors. An important property of quorum sensing communication is that the response is only achieved after one specific signal (i.e., cell number) threshold is exceeded. The response is mediated by a positive feedback loop of auto-inducer production, since genes for the enzymes that biosynthesize the signals are under their own control. There is a plethora of behaviors and phenotypes controlled by quorum sensing systems, including light production by several species of the *Vibrio* genus, competence (i.e., the ability to uptake and incorporate foreign DNA), biofilm formation, synthesis of secondary metabolites and the production of virulence factors.

Self-awareness can be described as the ability to recognize oneself as an individual separate from the environment and other individuals. Quorum sensing provides the entire bacterial network with the ability to recognize and adjust itself collectively once a specific population threshold is exceeded. This is specific for all individuals of a certain organism and even strain. Quorum sensing, therefore, can be viewed as a kind of self-awareness among isogenic bacterial populations.

Signaling related to specific environmental cues is interwoven with quorum sensing signaling; for example in *P. aeruginosa*, the iron availability signal network and the quorum sensing system communicate and influence each other (Juhas et al., 2004). In addition, bacteria can sense quorum sensing signals of other species (Federle, 2009) and act in accordance with the population sizes of competing or mutualistic species, including cells of eukaryotic or pluricellular organisms, such as their hosts (Bansal et al., 2010; Hosni et al., 2011; Ma et al., 2012). Thus, microbial networks have the ability to distinguish themselves from similar networks in other species. Most of the bacterial cell–cell communication described to date exclusively involves the release of autoinducers to the extracellular medium and the sensing of those molecules by other cells; phenomena that depend on the diffusion of signals and therefore lack directionality. Since, in a well-mixed environment such as a stirred liquid culture of planktonic cells, one cell can sense the auto-inducer produced by any other cell, communication among network components should be uniform. This is in contrast to communication among molecule types in signal transduction networks and among cells in neuronal networks. In the latter cases, each member interacts directly with a limited set of other network components, creating clusters and functional domains that, together, form a structured network with non-trivial topological features and a higher-than-random complexity. The situation changes in more realistic environments, such as in bacterial biofilms, which are known to be the preferred lifestyle of several bacterial species (Costerton et al., 1995). Those biofilms can be composed of a single bacterium species, but more often are complex ecologies of single-cell organisms that may include hundreds of different species of algae, bacteria, protozoa, fungi and viruses. They collectively generate and embed themselves in an extracellular polymeric matrix that provides structure and protection. In such environments, cell–cell communication could be more specifically performed among clusters of cells organized in different spatial and functional biofilm domains. Recently, the discovery of bacterial communication networks of multiple cells of *B. subtilis* that are directly connected to others by bacterial nanotubes was reported (Dubey and Ben-Yehuda, 2011). These structures are able to mediate the exchange of non-conjugative plasmids, metabolites and even enzymes, and can be formed in an interspecies manner between *B. subtilis* and *Staphylococcus aureus* or even the phylogenetically more distant *E. coli*. The authors speculated that these kinds of networks may represent a major form of bacterial communication in nature. If so, they may constitute complex and intricate structured bacterial communication networks with high potential to exhibit intelligent behavior.

Some features of self-awareness can be manifested already at a lower level of social organization of microorganisms. Thus, bacteria of the same species are capable of assembling together and isolating themselves from other species. This advanced social organization would be reflected in cooperation; for example in swarming motility (coordinated translocation of many bacterial cells), in collective repairing of holes in biofilm, in collective capture and digestion of food, etc. Microorganisms can cooperate for collective aggression through the coordinated production of antibiotics. There are even “bacteria-altruists,” who sacrifice themselves to become food for their brethren (Oleskin, 2009). However, at the opposite extreme, there also exist “microbe-cheaters,” which can disrupt cooperative systems by acquiring a disproportionate share of group-generated resources while making relatively small contributions (Velicer, 2003).

Gram negative bacterial pathogens, such as *P. aeruginosa*, *E. coli* enteropathogenic strains and several *Vibrio* species, and Gram positive pathogens, such as *S. aureus*, use QS to coordinate expression of several virulence determinants (Antunes et al., 2010). Beyond prokaryotes, QS is also used by eukaryotic pathogens, like the fungi *Candida albicans* (Nickerson et al., 2006), and even more complex microbes, such as parasitic protozoa like *Trypanosoma brucei* (Mony et al., 2014).

Although QS systems have been studied mostly in microbial pathogens, it has been discovered recently that several harmless free-living bacteria, such as cyanobacteria (Sharif et al., 2008; Zhai et al., 2012) and methanogenic Archaea (Zhang et al., 2012), also possess QS communication systems. Unlike pathogenic organisms, however, these microbes appear to use QS to achieve robust adaptation to environmental change. This is accomplished by redirecting metabolic fluxes at high cellular densities to optimize energy and resource consumption. In this sense, QS allows communities of related microbes to anticipate and prepare for nutrient scarcity (Sharif et al., 2008; Zhang et al., 2012). QS may even play a key role in establishing biofilms and initiating cellular blooms of cyanobacteria (Zhai et al., 2012).

In free living bacteria, QS contributes to cell differentiation and establishment of multicellular populations. A classic example of QS-mediated cell differentiation in bacteria is starvation-induced reproductive fruiting body development in myxobacteria. In *Myxococcus xanthus*, for example, soluble quorum-sensing A-signal assesses starvation and mediates the initial stages of cell aggregation (Kaiser, 2004). Furthermore, filamentous cyanobacteria exhibit one of the most complex cell differentiation processes observed in bacteria. These microbes can differentiate into four different cell types, including: (i) multicellular filaments that branch in multiple dimensions (trichomes); (ii) specialized nitrogen fixing cells called heterocysts; (iii) spore-like cells called akinetes; and (iv) hormogonia, which are small motile filaments that are important for dispersal (Flores and Herrero, 2010; Schirrmeister et al., 2011). So far, calcium cell signaling has been implicated in development of heterocysts (Torrecilla et al., 2004). Given that QS was recently discovered in these organisms (Sharif et al., 2008), it will be interesting to see what, if any, role QS plays in these differentiation pathways. Multicellularity, even in microbial populations, is an adaptation that allows cells to perform complex tasks and exhibit intelligent behaviors, like coordinating community-wide responses to environmental change. QS clearly plays a role in establishing multicellularity in microbes, but may also be the chemical language for communication of that intelligence.

The complexity of bacterial biofilms is equally striking. These rich ecosystems provide an environment for microbes to demonstrate their individual and collective intelligences. The human oral cavity, for example, contains hundreds of different bacterial, viral and fungal species. These species establish complex relationships, including both competitive and cooperative behaviors. We call the biofilm formed by these microbes the “dental plaque.” While many plaque species are commensal, some may become pathogenic in response to environmental triggers. A sudden shift in biofilm composition or dynamics may lead to dental caries and several other periodontal diseases (Avila et al., 2009). Among the dental plaque residents, *Porphyromonas gingivalis* is of particular concern. This species is a predominant contributor to human periodontitis. It employs several intricate mechanisms to subvert the innate immune system of the host. In fact, these evasive strategies are so clever that they have been compared to military tactics used in “guerilla” wars (Hajishengallis, 2009). Complex microbial communities are located in the gut of mammals as well. These highly dynamic, species-rich communities help modulate the host’s immune system. They are implicated in several human diseases, including chronic inflammatory diseases, such as Crohn’s disease (Macfarlane et al., 2011; Clemente et al., 2012), as well as obesity (Ridaura et al., 2013) and diabetes (Everard and Cani, 2013). Some evidence even suggests that microbes may alter human brain function and behavior (Cryan and Dinan, 2012). The ability of the microbiome to influence human intelligence has earned it the title, “the forgotten organ” (Relman and Falkow, 2001). Together, these results suggest that symbiotic microbiota may have played an important role in the evolution of plants and animals, leading some to contend that the unit of selection in evolution may be the holobiont, i.e., “the animal or plant with all of its associated microorganisms” (Zilber-Rosenberg and Rosenberg, 2008).

Finally, it is worthwhile to note that philosophers of biology are beginning to appreciate the remarkable microbial capacities for cooperation and communication (O’Malley and Dupré, 2007a; O’Malley, 2007b; O’Malley, 2013).

### PROBLEM SOLVING

An essential feature of any intelligent system is that, in addition to storing information and incorporating new knowledge from experiences, it must have the ability to use that knowledge to solve new problems. Generally, the more complex a problem a system can solve, the more intelligent it is considered. In this regard, some microorganism networks show problem solving abilities that can even match or surpass those shown by human beings: the slime mold *Physarum polycephalum* in its plasmodium configuration – a large multi-nuclear amoeba-like cell consisting of a dendritic network of pseudopodia – has the ability to connect two different food sources located at different points using the minimum-length pathway in a labyrinth, which optimizes its foraging efficiency (Nakagaki et al., 2000). The mold is able to create solutions with comparable efficiency, fault tolerance and cost to those of human infrastructure networks, such as the Tokyo rail system, but, unlike humans, the mold achieves optimal solutions solely by a process of selective reinforcement of the preferred routes and the simultaneous removal of redundant connections, without any centralized control or explicit global information. This striking mold ability was captured in a mathematical model, which the authors claim can provide a starting point to improve routing protocols and topology control for self-organized networks used for human transport and communication systems (Tero et al., 2010). This is a perfect example of applied microbial intelligence with the potential to improve human engineering.

## LEARNING FROM INTELLIGENCE IN THE MICROBIAL WORLD

Given the examples of the previous section, it is likely that, at least for some specific tasks, microbial “intelligence” can be compared to human intelligence, and microbial networks could be considered formally as “intelligent.” Recognizing microbial intelligence can allow us to potentially modify microbial networks or to develop new microbial networks capable of intelligent solutions to specific human problems *de novo*. If intelligence (or components thereof) emerges from the dynamics of complex adaptive systems and the human brain is an evolved organ for the encapsulation of intelligent characteristics, it is possible that there are features of intelligence that remain undiscovered.

### A DEEPER UNDERSTANDING OF THE MICROBIAL WORLD

One important and exciting domain of synthetic biology is the manipulation and design of microbial metabolism for chemical production in the energy, biomedicine and food industry (Purnick and Weiss, 2009). Such design relies on effective control and adaptation of metabolism (e.g., pathway flux) in response to intracellular or environmental perturbations. In an engineered genetic-metabolic circuit, there are many parameters that can be used for design purposes. Promoter characteristics, such as tightness, strength or regulatory sites, can be engineered in the transcriptional control, and the engineering of ribosome binding sites or RNA degradation can be used to control the expression levels of proteins. Well-known examples are the genetic control of lycopene production in *E. coli* (Farmer and Liao, 2000) and the design of gene-metabolic oscillators (Fung et al., 2005; Stricker et al., 2008). Designing scaffold proteins in the protein–protein interaction domain has been studied for the control of metabolic flux (Dueber et al., 2009). Recent studies (He et al., 2013; Westerhoff et al., 2014) showed that although gene-expression regulation can increase the robustness of an intermediate metabolite concentration, it rarely makes the metabolic pathway infinitely robust. For perfect adaptation to occur, the protein degradation reactions should be zero-order in the concentration of the protein or the living cell should enter stationary phase after a period of growth. The former scenario is rarely observed biologically; nevertheless, in some situations, protein degradation rates can be controlled by adding or removing a degradation tag to the gene sequence (McGinness et al., 2006). In this way, a relatively small degradation rate may be obtained in an engineered gene-metabolic network, and near-perfect adaptation behavior can be achieved with a quasi-integral control structure.

### MICROBIAL VS. HUMAN INTELLIGENCE

Our paper collects various examples of the intelligent features discovered in the microbial world (**Figure 1**). Microbial intelligence emerges from the dynamic interactions among macromolecules. Intelligence is a strong form of emergence; its reconstruction requires information of state-dependent component properties. The more state-dependent information we need, the stronger the emergence is. The degree of state-dependency of the component property is determined by the presence of other components in the system affecting this property, on the flux of matter through the system and on the history of the system (Kolodkin et al., 2012a,b, 2013b). In this context, we can scale and compare the strength of emergence of intelligence for different complex adaptive systems, e.g., for microorganisms, animals or humans.

In bacteria, there are many potential intracellular interactions that can affect the state-dependent property of a certain molecule. For example, the ability of a single transcription factor to bind a response element might depend on the presence of other transcription factors and their ligands, on components involved in intracellular trafficking of these ligands, on molecules providing ATP-convertible free energy for this trafficking and for receptor synthesis and even on molecules maintaining pH, viscosity, macromolecular crowding, etc. Thus, the emergence of intelligence that is raised due to interactions in an intracellular microbial network can be very strong indeed. On the other hand, the number of neurons affecting the firing of a single neuron in the human brain is tremendously high; and this is before we consider the intracellular interactions occurring in each and every neuronal cell, all of which contribute to the strength of the emergence of intelligence in our brains. Are these intelligences even comparable? We intuitively feel that the intelligence in microorganisms and in humans is different.

The physiological adaptive behavior of microorganisms is not stable and disappears when the environment does not support this behavior. Programs of adaptive behavior are imprinted on the population genome. When adaptation is lost, new training is required to regain this adaptation. Microorganisms exhibit some features of elastic behavior, but they do not have the conditional reflexes of higher animals. In an evolutionary context, in animals the elementary reflection of the environment is replaced by perceptive reflection and animals gain different forms of individually adapted behavioral changes co-tuned to the changes in the environment. Animal activity toward objects develops depending on the objects animals have already dealt with. This correlates with anatomical changes; the cerebral cortex emerges in addition to basal ganglia that cause a crucial shift in animal behavior. Basal ganglia enable signal reception and turn on inherited behavioral programs. The cerebral cortex, in its turn, enables analysis and integration of external signals, reflection on external objects and situations, building up of new connections and, ultimately, development of the behavior that is based, not on the inherited programs, but rather on the animal’s perception of external reality. With the development of the cerebral cortex, new forms of individual behavior based on objective reflection of the environment are formed.

Further development of the cerebral cortex takes place in humans. Aside from both inherited programs and individually gained experience, humans develop a third form of behavior: the ability to transfer collective experience from one human being to another. The transfer of collective experience includes the knowledge gained at school, at work, in life, etc. Animals are born with the inherited programs and enrich these programs through individual experience. Humans might be born with the poorest instinctive inborn programs, but can develop their mental processes, not only through personal experience, but also through learning from collective experience. Human individuals are able to communicate with each other and even, through the media of oral tradition and written history, with their predecessors. Nevertheless, in the context of scaling the degree of the strength of emergence, the complexity of the human brain does not change immensely compared to the brain of an animal. Rather, the new behavior emerges from the changes in the design, and not from a tremendous increase of interacting components.

Intelligence is a strongly emergent property in both microorganisms and animals, including humans. Still, there is a difference in the way these intelligences are manifested. Thus, humans study microorganisms and debate about microbial intelligence, and bacteria, while supremely adapted and aware of their environments, are probably not even aware of us and our endeavors.

### THE WAY FORWARD

Most aspects of human intelligence are also exhibited by microorganisms at least to some degree, except those that depend on reading, writing and listening. The examples we presented regarding quorum sensing and problem solving were from multicellular networks. The question remains whether networks at any single, more molecular level, such as intracellular signaling, also exhibit most aspects of intelligence. It has been proposed that intracellular quorum sensing occurs during mitochondrial apoptosis (Brady et al., 2006). The hierarchy of regulatory networks involved in ammonia assimilation is a candidate for rich intelligent behavior. The molecular information is now so complete (van Heeswijk et al., 2013) that it may well be possible to develop the existing replica models (Bruggeman et al., 2005) into a full representation. These may then be used to determine the extent to which our present molecular network understanding suffices to demonstrate that these networks should be expected to exhibit almost all types of intelligent behavior (Hellingwerf et al., 1995; Bruggeman et al., 2000). This could then also help with experimental design driving subsequent experimental testing. Similarly, such mathematical representations may also be used to search for new aspects of intelligence that we, as humans, do not recognize as such, for example adjustable robustness, random creativity facilitated by deterministic chaos in the networks, productive noise thereby, and read-only memory. Many of these aspects may be useful for synthetic biology; a synthetic biology that will give rise to much more sustainable, productive systems.

## Conflict of Interest Statement

The authors declare that the research was conducted in the absence of any commercial or financial relationships that could be construed as a potential conflict of interest.

## References

[B1] Ahrens M. B. Orger M. B. Robson D. N. Li J. M. Keller P. J. (2013). Whole-brain functional imaging at cellular resolution using light-sheet microscopy. *Nat. Methods* 10 413–420 10.1038/nmeth.2434 23524393

[B2] Albanesi D. Reh G. Guerin M. E. Schaeffer F. Debarbouille M. Buschiazzo A. (2013). Structural basis for feed-forward transcriptional regulation of membrane lipid homeostasis in *Staphylococcus aureus*. *PLoS Pathog.* 9:e1003108 10.1371/journal.ppat.1003108 PMC3536700 23300457

[B3] Alberghina L. Westerhoff H. V. (eds). (2005). *Systems Biology: Definitions and Perspectives*. Berlin: Springer 10.1007/b95175

[B4] Alivisatos A. P. Chun M. Church G. M. Greenspan R. J. Roukes M. L. Yuste R. (2012). The brain activity map project and the challenge of functional connectomics. *Neuron* 74 970–974 10.1016/j.neuron.2012.06.006 22726828 PMC3597383

[B5] Alon U. (2007). Network motifs: theory and experimental approaches. *Nat. Rev. Genet.* 8 450–461 10.1038/nrg2102 17510665

[B6] Alon U. Surette M. G. Barkai N. Leibler S. (1999). Robustness in bacterial chemotaxis. *Nature* 397 168–171 10.1038/16483 9923680

[B7] Antunes L. C. Ferreira R. B. Buckner M. M. Finlay B. B. (2010). Quorum sensing in bacterial virulence. *Microbiology* 156 2271–2282 10.1099/mic.0.038794-0 20488878

[B8] Arkin A. Ross J. Mcadams H. H. (1998). Stochastic kinetic analysis of developmental pathway bifurcation in phage lambda-infected *Escherichia coli* cells. *Genetics* 149 1633–1648 9691025 10.1093/genetics/149.4.1633 PMC1460268

[B9] Assadi S. M. Yucel M. Pantelis C. (2009). Dopamine modulates neural networks involved in effort-based decision-making. *Neurosci. Biobehav. Rev.* 33 383–393 10.1016/j.neubiorev.2008.10.010 19046987

[B10] Avila M. Ojcius D. M. Yilmaz O. (2009). The oral microbiota: living with a permanent guest. *DNA Cell Biol.* 28 405–411 10.1089/dna.2009.0874 19485767 PMC2768665

[B11] Bansal T. Alaniz R. C. Wood T. K. Jayaraman A. (2010). The bacterial signal indole increases epithelial-cell tight-junction resistance and attenuates indicators of inflammation. *Proc. Natl. Acad. Sci. U.S.A.* 107 228–233 10.1073/pnas.0906112107 19966295 PMC2806735

[B12] Ben Jacob E. Becker I. Shapira Y. Levine H. (2004). Bacterial linguistic communication and social intelligence. *Trends Microbiol.* 12 366–372 10.1016/j.tim.2004.06.006 15276612

[B13] Berg H. C. Tedesco P. M. (1975). Transient-response to chemotactic stimuli in *Escherichia coli*. *Proc. Natl. Acad. Sci. U.S.A.* 72 3235–3239 10.1073/pnas.72.8.3235 1103143 PMC432957

[B14] Berry D. B. Guan Q. N. Hose J. Haroon S. Gebbia M. Heisler L. E. (2011). Multiple means to the same end: the genetic basis of acquired stress resistance in yeast. *PLoS Genet.* 7:e1002353 10.1371/journal.pgen.1002353 PMC3213159 22102822

[B15] Bonneau R. Facciotti M. T. Reiss D. J. Schmid A. K. Pan M. Kaur A. (2007). A predictive model for transcriptional control of physiology in a free living cell. *Cell* 131 1354–1365 10.1016/j.cell.2007.10.053 18160043

[B16] Boogerd F. C. Bruggeman F. J. Richardson R. C. (2013). Mechanistic explanations and models in molecular systems biology. *Found. Sci.* 18 725–744 10.1007/s10699-012-9302-y

[B17] Boogerd F. C. Bruggeman F. J. Richardson R. C. Stephan A. Westerhoff H. V. (2005). Emergence and its place in nature: a case study of biochemical networks. *Synthese* 145 131–164 10.1007/s11229-004-4421-9

[B18] Boogerd F. C. Ma H. Bruggeman F. J. Van Heeswijk W. C. Garcia-Contreras R. Molenaar D. (2011). AmtB-mediated NH3 transport in prokaryotes must be active and as a consequence regulation of transport by GlnK is mandatory to limit futile cycling of NH4+/NH3. *FEBS Lett.* 585 23–28 10.1016/j.febslet.2010.11.055 21134373

[B19] Bourret R. B. Stock A. M. (2002). Molecular information processing: lessons from bacterial chemotaxis. *J. Biol. Chem.* 277 9625–9628 10.1074/jbc.R100066200 11779877

[B20] Brady N. R. Hamacher-Brady A. Westerhoff H. V. Gottlieb R. A. (2006). A wave of reactive oxygen species (ROS)-induced ROS release in a sea of excitable mitochondria. *Antioxid. Redox. Signal.* 8 1651–1665 10.1089/ars.2006.8.1651 16987019

[B21] Brooks A. N. Turkarslan S. Beer K. D. Lo F. Y. Baliga N. S. (2011). Adaptation of cells to new environments. *Wiley Interdiscip. Rev. Syst. Biol. Med.* 3 544–561 10.1002/wsbm.136 21197660 PMC3081528

[B22] Brooks R. A. (1991). Intelligence without representation. *Artif. Intell.* 47 139–159 10.1016/0004-3702(91)90053-M

[B23] Bruggeman F. J. Boogerd F. C. Westerhoff H. V. (2005). The multifarious short-term regulation of ammonium assimilation of *Escherichia coli*: dissection using an in silico replica. *FEBS J.* 272 1965–1985 10.1111/j.1742-4658.2005.04626.x 15819889

[B24] Bruggeman F. J. Van Heeswijk W. C. Boogerd F. C. Westerhoff H. V. (2000). Macromolecular intelligence in microorganisms. *Biol. Chem.* 381 965–972 10.1515/Bc.2000.119 11076029

[B25] Carrera J. Elena S. F. Jaramillo A. (2012). Computational design of genomic transcriptional networks with adaptation to varying environments. *Proc. Natl. Acad. Sci. U.S.A.* 109 15277–15282 10.1073/pnas.1200030109 22927389 PMC3458320

[B26] Clark K. B. (2010a). Arrhenius-kinetics evidence for quantum tunneling in microbial “social” decision rates. *Commun. Integr. Biol.* 3 540–544 10.4161/cib.3.6.12842 21331234 PMC3038058

[B27] Clark K. B. (2010b). On classical and quantum error-correction in ciliate mate selection. *Commun. Integr. Biol.* 3 374–378 10.4161/cib.3.4.11974 20798831 PMC2928323

[B28] Clark K. B. (2010c). Origins of learned reciprocity in solitary ciliates searching grouped ‘courting’ assurances at quantum efficiencies. *Biosystems* 99 27–41 10.1016/j.biosystems.2009.08.005 19686801

[B29] Clark K. B. (2012). Social biases determine spatiotemporal sparseness of ciliate mating heuristics. *Commun. Integr. Biol.* 5 3–11 10.4161/cib.18337 22482001 PMC3291309

[B30] Clark K. B. (2013). Ciliates learn to diagnose and correct classical error syndromes in mating strategies. *Front. Microbiol.* 4:229 10.3389/fmicb.2013.00229 PMC3746415 23966987

[B31] Clemente J. C. Ursell L. K. Parfrey L. W. Knight R. (2012). The impact of the gut microbiota on human health: an integrative view. *Cell* 148 1258–1270 10.1016/j.cell.2012.01.035 22424233 PMC5050011

[B32] Corning W. C. Von Burg R. (1973). “Protozoa,” in *Invertebrate Learning* eds Corning W. C. Dyal J. A. Willows A. O. D. (New York, NY: Plenum Press) 49–122 10.1007/978-1-4684-3006-6_2

[B33] Costerton J. W. Lewandowski Z. Caldwell D. E. Korber D. R. Lappin-Scott H. M. (1995). Microbial biofilms. *Annu. Rev. Microbiol.* 49 711–745 10.1146/annurev.mi.49.100195.003431 8561477

[B34] Cryan J. F. Dinan T. G. (2012). Mind-altering microorganisms: the impact of the gut microbiota on brain and behaviour. *Nat. Rev. Neurosci.* 13 701–712 10.1038/nrn3346 22968153

[B35] Csete M. E. Doyle J. C. (2002). Reverse engineering of biological complexity. *Science* 295 1664–1669 10.1126/science.1069981 11872830

[B36] Dubey G. P. Ben-Yehuda S. (2011). Intercellular nanotubes mediate bacterial communication. *Cell* 144 590–600 10.1016/j.cell.2011.01.015 21335240

[B37] Dueber J. E. Wu G. C. Malmirchegini G. R. Moon T. S. Petzold C. J. Ullal A. V. (2009). Synthetic protein scaffolds provide modular control over metabolic flux. *Nat. Biotechnol.* 27 753–759 10.1038/nbt.1557. 19648908

[B38] Dumas Z. Ross-Gillespie A. Kummerli R. (2013). Switching between apparently redundant iron-uptake mechanisms benefits bacteria in changeable environments. *Proc. Biol. Sci.* 280 20131055 10.1098/rspb.2013.1055 PMC3712426 23760867

[B39] El-Samad H. Goff J. P. Khammash M. (2002). Calcium homeostasis and parturient hypocalcemia: an integral feedback perspective. *J. Theor. Biol.* 214 17–29 10.1006/jtbi.2001.2422 11786029

[B40] Everard A. Cani P. D. (2013). Diabetes, obesity and gut microbiota. *Best Pract. Res. Clin. Gastroenterol.* 27 73–83 10.1016/j.bpg.2013.03.007 23768554

[B41] Farmer W. R. Liao J. C. (2000). Improving lycopene production in *Escherichia coli* by engineering metabolic control. *Nat. Biotechnol.* 18 533–537 10.1038/75398 10802621

[B42] Federle M. J. (2009). Autoinducer-2-based chemical communication in bacteria: complexities of interspecies signaling. *Contrib. Microbiol.* 16 18–32 10.1159/000219371 19494577 PMC3042238

[B43] Fell D. A. (1997). *Understanding the Control of Metabolism*. London: Portland Press.

[B44] Feynman R. P. (1982). Simulating physics with computers. *Int. J. Theor. Phys.* 21 467–488 10.1007/bf02650179

[B45] Flores E. Herrero A. (2010). Compartmentalized function through cell differentiation in filamentous cyanobacteria. *Nat. Rev. Microbiol.* 8 39–50 10.1038/nrmicro2242 19966815

[B46] Fung E. Wong W. W. Suen J. K. Bulter T. Lee S. G. Liao J. C. (2005). A synthetic gene-metabolic oscillator. *Nature* 435 118–122 10.1038/nature03508 15875027

[B47] Gelber B. (1952). Investigations of the behavior of Paramecium aurelia. I. Modification of behavior after training with reinforcement. *J. Comp. Physiol. Psychol.* 45 58–65 10.1037/h0063093 14907934

[B48] Gleichgerrcht E. Ibanez A. Roca M. Torralva T. Manes F. (2010). Decision-making cognition in neurodegenerative diseases. *Nat. Rev. Neurol.* 6 611–623 10.1038/nrneurol.2010.148 21045795

[B49] Goo E. Majerczyk C. D. An J. H. Chandler J. R. Seo Y. S. Ham H. (2012). Bacterial quorum sensing, cooperativity, and anticipation of stationary-phase stress. *Proc. Natl. Acad. Sci. U.S.A.* 109 19775–19780 10.1073/pnas.1218092109 23150539 PMC3511722

[B50] Gorbunova K. O. (1999). “Kinetic model of parallel data processing,” in *Parallel Computing Technologies* ed. Malyshkin V. (Berlin, Heidelberg: Springer) 54–59

[B51] Hajishengallis G. (2009). Porphyromonas gingivalis–host interactions: open war or intelligent guerilla tactics? *Microbes Infect.* 11 637–645 10.1016/j.micinf.2009.03.009 19348960 PMC2704251

[B52] Hansen C. H. Endres R. G. Wingreen N. S. (2008). Chemotaxis in *Escherichia coli*: a molecular model for robust precise adaptation. *PLoS Comput. Biol.* 4:e1 10.1371/journal.pcbi.0040001 PMC2174977 18179279

[B53] Hao N. Behar M. Elston T. C. Dohlman H. G. (2007). Systems biology analysis of G protein and MAP kinase signaling in yeast. *Oncogene* 26 3254–3266 10.1038/sj.onc.1210416 17496920

[B54] He F. Fromion V. Westerhoff H. V. (2013). (Im)Perfect robustness and adaptation of metabolic networks subject to metabolic and gene-expression regulation: marrying control engineering with metabolic control analysis. *BMC Syst. Biol.* 7:131 10.1186/1752-0509-7-131 PMC4222491 24261908

[B55] Hellingwerf K. J. Postma P. W. Tommassen J. Westerhoff H. V. (1995). Signal-transduction in bacteria – phospho-neural network(s) in *Escherichia coli*. *FEMS Microbiol. Rev.* 16 309–321 10.1111/j.1574-6976.1995.tb00178.x 7654406

[B56] Hermundstad A. M. Brown K. S. Bassett D. S. Carlson J. M. (2011). Learning, memory, and the role of neural network architecture. *PLoS Comput. Biol.* 7:e1002063 10.1371/journal.pcbi.1002063 PMC3127797 21738455

[B57] Hinkle D. J. Wood D. C. (1994). Is tube-escape learning by protozoa associative learning? *Behav. Neurosci.* 108 94–99 10.1037/0735-7044.108.1.94 8192854

[B58] Hoffer S. M. Westerhoff H. V. Hellingwerf K. J. Postma P. W. Tommassen J. (2001). Autoamplification of a two-component regulatory system results in “learning” behavior. *J. Bacteriol.* 183 4914–4917 10.1128/Jb.183.16.4914-4917.2001 11466297 PMC99548

[B59] Hopfield J. J. (1982). Neural networks and physical systems with emergent collective computational abilities. *Proc. Natl. Acad. Sci. U.S.A.* 79 2554–2558 10.1073/pnas.79.8.2554 6953413 PMC346238

[B60] Hosni T. Moretti C. Devescovi G. Suarez-Moreno Z. R. Fatmi M. B. Guarnaccia C. (2011). Sharing of quorum-sensing signals and role of interspecies communities in a bacterial plant disease. *ISME J.* 5 1857–1870 10.1038/ismej.2011.65 21677694 PMC3223305

[B61] Jarrell K. F. McBride M. J. (2008). The surprisingly diverse ways that prokaryotes move. *Nat. Rev. Microbiol.* 6 466–476 10.1038/nrmicro1900 18461074

[B62] Jensen D. D. (1957). Experiments on learning in paramecia. *Science* 125 191–192 10.1126/science.125.3240.191 13390982

[B63] Juhas M. Wiehlmann L. Huber B. Jordan D. Lauber J. Salunkhe P. (2004). Global regulation of quorum sensing and virulence by VqsR in *Pseudomonas aeruginosa*. *Microbiology* 150 831–841 10.1099/mic.0.26906.0 15073293

[B64] Kahn D. Westerhoff H. V. (1991). Control theory of regulatory cascades. *J. Theor. Biol.* 153 255–285 10.1016/S0022-5193(05)80426-6 1686290

[B65] Kaiser D. (2004). Signaling in myxobacteria. *Annu. Rev. Microbiol.* 58 75–98 10.1146/annurev.micro.58.030603.123620 15487930

[B66] Kamp F. Westerhoff H. V. (1986). “Molecular machines and energy channelling,” in *The Organization of Cell Metabolism* eds Welch G. R. Clegg J. S. 1 (London: Plenum Press).

[B67] Kashtan N. Itzkovitz S. Milo R. Alon U. (2004). Topological generalizations of network motifs. *Phys. Rev. E Stat. Nonlin. Soft Matter Phys.* 70:031909 10.1103/Physreve.70.031909 15524551

[B68] Kolodkin A. Boogerd F. C. Plant N. Bruggeman F. J. Goncharuk V. Lunshof J. (2012a). Emergence of the silicon human and network targeting drugs. *Eur. J. Pharm. Sci.* 46 190–197 10.1016/j.ejps.2011.06.006 21704158

[B69] Kolodkin A. Simeonidis E. Balling R. Westerhoff H. (2012b). Understanding complexity in neurodegenerative diseases: in silico reconstruction of emergence. *Front. Physiol.* 3:291 10.3389/fphys.2012.00291 PMC3429063 22934043

[B70] Kolodkin A. Sahin N. Phillips A. Hood S. R. Bruggeman F. J. Westerhoff H. V. (2013a). Optimization of stress response through the nuclear receptor-mediated cortisol signalling network. *Nat. Commun.* 4:1792 10.1038/Ncomms2799 PMC3644104 23653204

[B71] Kolodkin A. Simeonidis E. Westerhoff H. V. (2013b). Computing life: add logos to biology and bios to physics. *Prog. Biophys. Mol. Biol.* 111 69–74 10.1016/j.pbiomolbio.2012.10.003 23103359

[B72] Kunita I. Kuroda S. Ohki K. Nakagaki T. (2014). Attempts to retreat from a dead-ended long capillary by backward swimming in *Paramecium*. *Front. Microbiol.* 5:270 10.3389/fmicb.2014.00270 PMC4052044 24966852

[B73] Lee J. T. Jayaraman A. Wood T. K. (2007). Indole is an inter-species biofilm signal mediated by SdiA. *BMC Microbiol.* 7:42 10.1186/1471-2180-7-42 PMC1899176 17511876

[B74] Leisner M. Stingl K. Frey E. Maier B. (2008). Stochastic switching to competence. *Curr. Opin. Microbiol.* 11 553–559 10.1016/j.mib.2008.09.020 18955155

[B75] Lengeler J. W. (2000). Metabolic networks: a signal-oriented approach to cellular models. *Biol. Chem.* 381 911–920 10.1515/BC.2000.112 11076022

[B76] Looijen R. C. (2000). *Holism and Reductionism in Biology and Ecology: The Mutual Dependence of Higher and Lower Level Research Programmes*. Dordrecht: Kluwer Academic Publishers. 10.1007/978-94-015-9560-5

[B77] Lyon P. (2006). The biogenic approach to cognition. *Cogn. Process.* 7 11–29 10.1007/s10339-005-0016-8 16628463

[B78] Ma Q. Fonseca A. Liu W. Fields A. T. Pimsler M. L. Spindola A. F. (2012). *Proteus mirabilis* interkingdom swarming signals attract blow flies. *ISME J.* 6 1356–1366 10.1038/ismej.2011.210 22237540 PMC3379643

[B79] Ma W. Z. Trusina A. El-Samad H. Lim W. A. Tang C. (2009). Defining network topologies that can achieve biochemical adaptation. *Cell* 138 760–773 10.1016/j.cell.2009.06.013 19703401 PMC3068210

[B80] Maamar H. Raj A. Dubnau D. (2007). Noise in gene expression determines cell fate in *Bacillus Subtilis*. *Science* 317 526–529 10.1126/science.1140818 17569828 PMC3828679

[B81] Macfarlane S. Bahrami B. Macfarlane G. T. (2011). Mucosal biofilm communities in the human intestinal tract. *Adv. Appl. Microbiol.* 75 111–143 10.1016/B978-0-12-387046-9.00005-0 21807247

[B82] Mahner M. Bunge M. (2001). Function and functionalism: a synthetic perspective. *Philos. Sci.* 68 75–94 10.1086/392867

[B83] Mangan S. Alon U. (2003). Structure and function of the feed-forward loop network motif. *Proc. Natl. Acad. Sci. U.S.A.* 100 11980–11985 10.1073/pnas.2133841100 14530388 PMC218699

[B84] Manin Y. I. (1980). *Vychislimoe i nevychislimoe.* Moscow: Sov. Radio

[B85] Mank N. N. Berghoff B. A. Klug G. (2013). A mixed incoherent feed-forward loop contributes to the regulation of bacterial photosynthesis genes. *RNA Biol.* 10 347–352 10.4161/Rna.23769 23392242 PMC3672276

[B86] Maren S. Phan K. L. Liberzon I. (2013). The contextual brain: implications for fear conditioning, extinction and psychopathology. *Nat. Rev. Neurosci.* 14 417–428 10.1038/Nrn3492 23635870 PMC5072129

[B87] McGinness K. E. Baker T. A. Sauer R. T. (2006). Engineering controllable protein degradation. *Mol. Cell.* 22 701–707 10.1016/j.molcel.2006.04.027 16762842

[B88] McGregor S. Vasas V. Husbands P. Fernando C. (2012). Evolution of associative learning in chemical networks. *PLoS Comput. Biol.* 8:e1002739 10.1371/journal.pcbi.1002739 PMC3486861 23133353

[B89] Mettetal J. T. Muzzey D. Gomez-Uribe C. Van Oudenaarden A. (2008). The frequency dependence of osmo-adaptation in *Saccharomyces cerevisiae*. *Science* 319 482–484 10.1126/science.1151582 18218902 PMC2916730

[B90] Milo R. Shen-Orr S. Itzkovitz S. Kashtan N. Chklovskii D. Alon U. (2002). Network motifs: simple building blocks of complex networks. *Science* 298 824–827 10.1126/science.298.5594.824 12399590

[B91] Mony B. M. Macgregor P. Ivens A. Rojas F. Cowton A. Young J. (2014). Genome-wide dissection of the quorum sensing signalling pathway in *Trypanosoma brucei*. *Nature* 505 681–685 10.1038/nature12864 24336212 PMC3908871

[B92] Muzzey D. Gomez-Uribe C. A. Mettetal J. T. Van Oudenaarden A. (2009). A systems-level analysis of perfect adaptation in yeast osmoregulation. *Cell* 138 160–171 10.1016/j.cell.2009.04.047 19596242 PMC3109981

[B93] Nakagaki T. Yamada H. Ueda T. (2000). Interaction between cell shape and contraction pattern in the Physarum plasmodium. *Biophys. Chem.* 84 195–204 10.1016/S0301-4622(00)00108-3 10852307

[B94] Nakazawa K. Quirk M. C. Chitwood R. A. Watanabe M. Yeckel M. F. Sun L. D. (2002). Requirement for hippocampal CA3 NMDA receptors in associative memory recall. *Science* 297 211–218 10.1126/science.1071795 12040087 PMC2877140

[B95] Neisser U. Boodoo G. Bouchard T. J. Boykin A. W. Brody N. Ceci S. J. (1996). Intelligence: knowns and unknowns. *Am. Psychol.* 51 77–101 10.1037/0003-066x.51.2.77

[B96] Nickerson K. W. Atkin A. L. Hornby J. M. (2006). Quorum sensing in dimorphic fungi: farnesol and beyond. *Appl. Environ. Microbiol.* 72 3805–3813 10.1128/AEM.02765-05 16751484 PMC1489610

[B97] Noirot P. Noirot-Gros M. F. (2004). Protein interaction networks in bacteria. *Curr. Opin. Microbiol.* 7 505–512 10.1016/j.mib.2004.08.005 15451506

[B98] O’Malley M. A. (2013). Philosophy and the microbe: a balancing act. *Biol. Philos.* 28 153–159 10.1007/s10539-013-9360-8

[B99] O’Malley M. A. Dupré J. (2007a). Size doesn’t matter: towards a more inclusive philosophy of biology. *Biol. Philos.* 22 155–191 10.1007/s10539-006-9031-0

[B100] O’Malley M. A. Dupré J. (2007b). Towards a philosophy of microbiology. *Stud. Hist. Philos. Biol. Biomed. Sci.* 38 775–779 10.1016/j.shpsc.2007.09.002 18053932

[B101] Oleskin A. V. (2009). Biosocial phenomena in unicellular organisms (exemplified by data concerning Prokaryota). *Zh. Obshch. Biol.* 70 225–238 19530599

[B102] Patnaik P. R. (2000). Are microbes intelligent beings? An assessment of cybernetic modeling. *Biotechnol. Adv.* 18 267–288 10.1016/S0734-9750(00)00037-9 14538104

[B103] Pavlov I. P. Anrep G. V. (1927). *Conditioned Reflexes: An Investigation of the Physiological Activity of the Cerebral Cortex*. London: Oxford University Press 10.5214/ans.0972-7531.1017309 PMC4116985 25205891

[B104] Purnick P. E. M. Weiss R. (2009). The second wave of synthetic biology: from modules to systems. *Nat. Rev. Mol. Cell Biol.* 10 410–422 10.1038/Nrm2698 19461664

[B105] Quinton-Tulloch M. J. Bruggeman F. J. Snoep J. L. Westerhoff H. V. (2013). Trade-off of dynamic fragility but not of robustness in metabolic pathways in silico. *FEBS J.* 280 160–173 10.1111/Febs.12057 23121761

[B106] Reiss D. J. Baliga N. S. Bonneau R. (2006). Integrated biclustering of heterogeneous genome-wide datasets for the inference of global regulatory networks. *BMC Bioinformatics* 7:280 10.1186/1471-2105-7-280 PMC1502140 16749936

[B107] Relman D. A. Falkow S. (2001). The meaning and impact of the human genome sequence for microbiology. *Trends Microbiol.* 9 206–208 10.1016/s0966-842x(01)02041-8 11336835

[B108] Ridaura V. K. Faith J. J. Rey F. E. Cheng J. Duncan A. E. Kau A. L. (2013). Gut microbiota from twins discordant for obesity modulate metabolism in mice. *Science* 341 1241214 10.1126/science.1241214 PMC3829625 24009397

[B109] Rolls E. T. Treves A. (1998). *Neural Networks and Brain Function.* Oxford: Oxford University Press

[B110] Rumpel S. Ledoux J. Zador A. Malinow R. (2005). Postsynaptic receptor trafficking underlying a form of associative learning. *Science* 308 83–88 10.1126/science.1103944 15746389

[B111] Salihoglu U. (2009). *Toward a Brain-like Memory with Recurrent Neural Networks.* Ph.D. thesis, Universite Libre de Bruxelles, Belgium

[B112] Sanger T. D. (1989). Optimal unsupervised learning in a single-layer linear feedforward neural network. *Neural Netw.* 2 459–473 10.1016/0893-6080(89)90044-0

[B113] Schirrmeister B. E. Antonelli A. Bagheri H. C. (2011). The origin of multicellularity in cyanobacteria. *BMC Evol. Biol.* 11:45 10.1186/1471-2148-11-45 PMC3271361 21320320

[B114] Sharif D. I. Gallon J. Smith C. J. Dudley E. (2008). Quorum sensing in Cyanobacteria: N-octanoyl-homoserine lactone release and response, by the epilithic colonial cyanobacterium Gloeothece PCC6909. *ISME J.* 2 1171–1182 10.1038/ismej.2008.68 18633449

[B115] Shudo E. Haccou P. Iwasa Y. (2003). Optimal choice between feedforward and feedback control in gene expression to cope with unpredictable danger. *J. Theor. Biol.* 223 149–160 10.1016/S0022-5193(03)00081-X 12814598

[B116] Smith S. L. Timmis J. (2008). An immune network inspired evolutionary algorithm for the diagnosis of Parkinson’s disease. *Biosystems* 94 34–46 10.1016/j.biosystems.2008.05.024 18619513

[B117] Snoep J. L. Van Der Weijden C. C. Andersen H. W. Westerhoff H. V. Jensen P. R. (2002). DNA supercoiling in *Escherichia coli* is under tight and subtle homeostatic control, involving gene-expression and metabolic regulation of both topoisomerase I and DNA gyrase. *Eur. J. Biochem.* 269 1662–1669 10.1046/j.1432-1327.2002.02803.x 11895436

[B118] Soest H. (1937). Dressurversuche mit Ciliaten und rhabdocoelen Turbellarien. *Z. Vgl. Physiol.* 24 720–748 10.1007/bf00592306

[B119] Sorek M. Balaban N. Q. Loewenstein Y. (2013). Stochasticity, bistability and the wisdom of crowds: a model for associative learning in genetic regulatory networks. *PLoS Comput. Biol.* 9:e1003179 10.1371/journal.pcbi.1003179 PMC3749950 23990765

[B120] Stephan A. (1999). Varieties of emergentism. *Evol. Cogn.* 5 49–59

[B121] Stephan A. (2006). The dual role of ‘emergence’ in the philosophy of mind and in cognitive science. *Synthese* 151 485–498 10.1007/s11229-006-9019-y

[B122] Stover C. K. Pham X. Q. Erwin A. L. Mizoguchi S. D. Warrener P. Hickey M. J. (2000). Complete genome sequence of *Pseudomonas aeruginosa* PAO1, an opportunistic pathogen. *Nature* 406 959–964 10.1038/35023079 10984043

[B123] St-Pierre F. Endy D. (2008). Determination of cell fate selection during phage lambda infection. *Proc. Natl. Acad. Sci. U.S.A.* 105 20705–20710 10.1073/pnas.0808831105 19098103 PMC2605630

[B124] Stricker J. Cookson S. Bennett M. R. Mather W. H. Tsimring L. S. Hasty J. (2008). A fast, robust and tunable synthetic gene oscillator. *Nature* 456 516–519 10.1038/nature07389 18971928 PMC6791529

[B125] Tagkopoulos I. Liu Y. C. Tavazoie S. (2008). Predictive behavior within microbial genetic networks. *Science* 320 1313–1317 10.1126/science.1154456 18467556 PMC2931280

[B126] Ter Kuile B. H. Westerhoff H. V. (2001). Transcriptome meets metabolome: hierarchical and metabolic regulation of the glycolytic pathway. *FEBS Lett.* 500 169–171 10.1016/S0014-5793(01)02613-8 11445079

[B127] Tero A. Nakagaki T. Toyabe K. Yumiki K. Kobayashi R. (2010). A method inspired by Physarum for solving the Steiner problem. *Int. J. Unconvent. Comput.* 6 109–123

[B128] Thorndike E. L. (1998). Animal intelligence – an experimental study of the associate processes in animals. *Am. Psychol.* 53 1125–1127 10.1037//0003-066x.53.10.1125

[B129] Torrecilla I. Leganes F. Bonilla I. Fernandez-Pinas F. (2004). A calcium signal is involved in heterocyst differentiation in the cyanobacterium *Anabaena* sp. PCC7120. *Microbiology* 150 3731–3739 10.1099/mic.0.27403-0 15528659

[B130] Trewavas A. (2002). Mindless mastery. *Nature* 415 841–841 10.1038/415841a 11859344

[B131] Turing A. M. (1950). Computing machinery and intelligence. *Mind* 433–460 10.1093/mind/LIX.236.433

[B132] van Duijn M. (2012). *The Biocognitive Spectrum: Biological Cognition as Variations on Sensorimotor Coordination*. Ph.D. thesis, University of Groningen, Netherlands

[B133] van Heeswijk W. C. Westerhoff H. V. Boogerd F. C. (2013). Nitrogen assimilation in *Escherichia coli*: putting molecular data into a systems perspective. *Microbiol. Mol. Biol. Rev.* 77 628–695 10.1128/MMBR.00025-13 24296575 PMC3973380

[B134] Veening J. W. Smits W. K. Kuipers O. P. (2008). Bistability, epigenetics, and bet-hedging in bacteria. *Annu. Rev. Microbiol.* 62 193–210 10.1146/annurev.micro.62.081307.163002 18537474

[B135] Velicer G. J. (2003). Social strife in the microbial world. *Trends Microbiol.* 11 330–337 10.1016/S0966-842x(03)00152-5 12875817

[B136] Waters C. M. Bassler B. L. (2005). Quorum sensing: cell-to-cell communication in bacteria. *Annu. Rev. Cell Dev. Biol.* 21 319–346 10.1146/annurev.cellbio.21.012704.131001 16212498

[B137] Wegrzyn G. Wegrzyn A. (2005). Genetic switches during bacteriophage lambda development. *Prog. Nucleic Acid Res. Mol. Biol.* 79 1–48 10.1016/S0079-6603(04)79001-7 16096026

[B138] Westerhoff H. V. (2008). Signalling control strength. *J. Theor. Biol.* 252 555–567 10.1016/j.jtbi.2007.11.035 18222488

[B139] Westerhoff H. V. Aon M. A. Van Dam K. Cortassa S. Kahn D. Van Workum M. (1990). Dynamic and hierarchical coupling. *Biochim. Biophys. Acta* 1018 142–146 10.1016/0005-2728(90)90235-V

[B140] Westerhoff H. V. He F. Murabito E. Crémazy F. Barberis M. (2014). “Understanding principles of the dynamic biochemical networks of life through systems biology,” in *Computational Systems Biology* 2nd Edn eds Kriete A. Eils R. (Oxford: Academic Press) 21–44

[B141] Westerhoff H. V. Kolodkin A. Conradie R. Wilkinson S. J. Bruggeman F. J. Krab K. (2009). Systems biology towards life in silico: mathematics of the control of living cells. *J. Math. Biol.* 58 7–34 10.1007/s00285-008-0160-8 18278498

[B142] Westerhoff H. V. Van Dam K. (1987). *Thermodynamics and Control of Biological Free Energy Transduction.* Amsterdam: Elsevier Science Ltd

[B143] Wouters A. G. (1995). Viability explanation. *Biol. Philos.* 10 435–457 10.1007/BF00857593

[B144] Wouters A. G. (1999). *Explanation without a Cause.* Utrecht: Zeno Institute of Philosophy

[B145] Wouters A. G. (2003). Four notions of biological function. *Stud. Hist. Philos. Biol. Biomed. Sci.* 34 633–668 10.1016/j.shpsc.2003.09.006

[B146] Wouters A. G. (2007). Design explanation: determining the constraints on what can be alive. *Erkenntnis* 67 65–80 10.1007/s10670-007-9045-2

[B147] Wouters A. G. (2013). “Biology’s functional perspective: roles, advantages and organization,” in *The Philosophy of Biology: A Companion for Educators* ed. Kampourakis K. (Netherlands: Springer) 455–486

[B148] Xavier J. B. (2011). Social interaction in synthetic and natural microbial communities. *Mol. Syst. Biol.* 7:483 10.1038/Msb.2011.16 PMC3101950 21487402

[B149] Yi T. M. Huang Y. Simon M. I. Doyle J. (2000). Robust perfect adaptation in bacterial chemotaxis through integral feedback control. *Proc. Natl. Acad. Sci. U.S.A.* 97 4649–4653 10.1073/pnas.97.9.4649 10781070 PMC18287

[B150] Zaborin A. Romanowski K. Gerdes S. Holbrook C. Lepine F. Long J. (2009). Red death in *Caenorhabditis elegans* caused by *Pseudomonas aeruginosa* PAO1. *Proc. Natl. Acad. Sci. U.S.A.* 106 6327–6332 10.1073/pnas.0813199106 19369215 PMC2669342

[B151] Zhai C. Zhang P. Shen F. Zhou C. Liu C. (2012). Does *Microcystis aeruginosa* have quorum sensing? *FEMS Microbiol. Lett.* 336 38–44 10.1111/j.1574-6968.2012.02650.x 22861498

[B152] Zhang G. Zhang F. Ding G. Li J. Guo X. Zhu J. (2012). Acyl homoserine lactone-based quorum sensing in a methanogenic archaeon. *ISME J.* 6 1336–1344 10.1038/ismej.2011.203 22237544 PMC3379639

[B153] Zilber-Rosenberg I. Rosenberg E. (2008). Role of microorganisms in the evolution of animals and plants: the hologenome theory of evolution. *FEMS Microbiol. Rev.* 32 723–735 10.1111/j.1574-6976.2008.00123.x 18549407

